# Goal-Directed Decision Making with Spiking Neurons

**DOI:** 10.1523/JNEUROSCI.2854-15.2016

**Published:** 2016-02-03

**Authors:** Johannes Friedrich, Máté Lengyel

**Affiliations:** ^1^Computational and Biological Learning Laboratory, Department of Engineering, University of Cambridge, Cambridge CB2 1PZ, United Kingdom, and; ^2^Department of Cognitive Science, Central European University, Budapest 1051, Hungary

**Keywords:** computational modeling, decision making, neuroeconomics, planning, reinforcement learning, spiking neurons

## Abstract

Behavioral and neuroscientific data on reward-based decision making point to a fundamental distinction between habitual and goal-directed action selection. The formation of habits, which requires simple updating of cached values, has been studied in great detail, and the reward prediction error theory of dopamine function has enjoyed prominent success in accounting for its neural bases. In contrast, the neural circuit mechanisms of goal-directed decision making, requiring extended iterative computations to estimate values online, are still unknown. Here we present a spiking neural network that provably solves the difficult online value estimation problem underlying goal-directed decision making in a near-optimal way and reproduces behavioral as well as neurophysiological experimental data on tasks ranging from simple binary choice to sequential decision making. Our model uses local plasticity rules to learn the synaptic weights of a simple neural network to achieve optimal performance and solves one-step decision-making tasks, commonly considered in neuroeconomics, as well as more challenging sequential decision-making tasks within 1 s. These decision times, and their parametric dependence on task parameters, as well as the final choice probabilities match behavioral data, whereas the evolution of neural activities in the network closely mimics neural responses recorded in frontal cortices during the execution of such tasks. Our theory provides a principled framework to understand the neural underpinning of goal-directed decision making and makes novel predictions for sequential decision-making tasks with multiple rewards.

**SIGNIFICANCE STATEMENT** Goal-directed actions requiring prospective planning pervade decision making, but their circuit-level mechanisms remain elusive. We show how a model circuit of biologically realistic spiking neurons can solve this computationally challenging problem in a novel way. The synaptic weights of our network can be learned using local plasticity rules such that its dynamics devise a near-optimal plan of action. By systematically comparing our model results to experimental data, we show that it reproduces behavioral decision times and choice probabilities as well as neural responses in a rich set of tasks. Our results thus offer the first biologically realistic account for complex goal-directed decision making at a computational, algorithmic, and implementational level.

## Introduction

Research in animal learning and behavioral neuroscience has given rise to the view that reward-based decision making is governed by (at least) two distinct strategies: a habit system, which relies on cached associations between actions or situations and their long-run future values, and a goal-directed system, which involves prospective planning and comparison of action outcomes based on an internal model of the environment (Daw et al., 2005). These systems have their formal counterparts in theories of model-free and model-based reinforcement learning, respectively. In particular, temporal-difference algorithms of model-free learning account for both behavioral and neuroimaging data regarding habit-based decision making (O'Doherty et al., 2004; Seymour et al., 2004). Similarly, model-based reinforcement learning algorithms have provided a powerful framework to account for goal-directed behavior and to identify some of the key brain areas involved in it (Gläscher et al., 2010; Daw et al., 2011). However, whereas the reward prediction error theory of the phasic responses of dopaminergic neurons has enjoyed prominent success in providing a circuit-level description of the implementation of model-free decision making (Schultz et al., 1997), much less is known about the neural circuit mechanisms of model-based decision making.

In the present work, we propose the first instantiation of model-based reinforcement learning in a biologically realistic network of spiking neurons. In particular, we show how such a network can compute a quantity that forms the basis of model-based decision making: the value of the best action in a given situation. This optimal value expresses the prediction of cumulative future reward after the execution of that action according to an internal model of how actions lead to future situations and rewards, and assuming that all later actions will also be chosen optimally so as to maximize the cumulative future reward. Thus, there is a strongly nonlinear, recursive relationship between the optimal values of different actions (formalized by the so-called Bellman optimality equation; Bellman, 1957), which makes the computation of optimal values challenging (Papadimitriou and Tsitsiklis, 1987). Indeed, goal-directed decision making is typically considered as flexible but slow, in contrast to inflexible but fast habitual decision making (Keramati et al., 2011).

We show that goal-directed planning is performed in our network on the time scale of hundreds of milliseconds in simpler one-step neuroeconomic, and even more complex sequential, decision-making tasks by using neurally plausible network dynamics. We first demonstrate that our network indeed competently solves the Bellman equation by establishing its performance on an example artificial decision-making task (after benchmarking it more extensively on a number of standard tasks from the reinforcement learning literature). We then present results from a set of simulations illustrating the success of our model at accounting for a variety of experimental findings involving goal-directed action selection. Our model reproduces behavioral and neurophysiological data on tasks ranging from simple binary choice to multistep sequential decision making. We also make predictions that are testable at the level of behavior and in neural responses for a new, multiple-reward variant of an already existing sequential decision-making task.

## Materials and Methods

### 

#### 

##### Optimal values.

Following the theory of Markov decision processes (MDPs), we formalize a sequential decision-making task as consisting of a number of states, or situations, in which the agent can be, *s*, in each of which there is a number of actions, *a*, available to the agent. Selecting action *a* in state *s* leads to another state, *s′*, with probability *P*(*s′*|*s*, *a*), and results in immediate, and potentially stochastic, reward *r* with expected value *r̄*(*s*, *a*). The goal of the agent is to select actions such that its cumulative expected reward over a long period is maximized.

The value of a state is the expected cumulative future reward once the agent passes through that state, and it is a central quantity for successful decision making that should guide the agent toward states with higher values. The Bellman equation establishes a recursive relationship for the values of the states when actions are chosen according to some particular “policy” (Sutton and Barto, 1998):

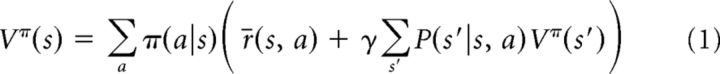
 where policy π(*a*|*s*) defines the probability with which action *a* is chosen in state *s*, and the temporal discounting factor 0 ≤ γ ≤ 1 formalizes the notion that future reward is worth less than immediate reward.

Under the optimal policy, which selects the best action in each state, the optimal values are related through the Bellman optimality equation (Sutton and Barto, 1998):

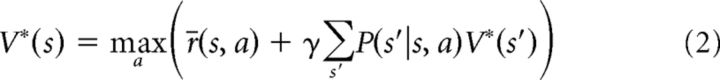
 which can be interpreted as the value of the best action in state *s*, assuming that actions are chosen optimally in all other states as well. Note that whereas Equation 1 expresses a linear relationship between values attainable by a particular policy, the recursive relationship between optimal values given by Equation 2 is strongly nonlinear (because of the max operator). Our goal is to show that a recurrently connected network of spiking neurons can compute and represent these optimal values through its internal dynamics.

##### Neural network dynamics.

We used a canonical simplified model of single-neuron dynamics, known as the stochastic spike–response model [Gerstner et al., 2014; or, equivalently, the generalized linear model (Truccolo et al., 2005)], which has proved to provide a compact but faithful description of neural responses in a number of cortical and subcortical areas (Pillow et al., 2005; Jolivet et al., 2006; 2008). According to this model, the (subthreshold) membrane potential of neuron *i*, *u*_i_ (measured with respect to the resting potential and excluding the waveform of action potentials), decays exponentially in the absence of inputs, whereas each presynaptic spike adds to it a constant waveform, ϵ(*t*), scaled by the corresponding synaptic weight, and each (postsynaptic) spike of the neuron itself is followed by instantaneous hyperpolarization:

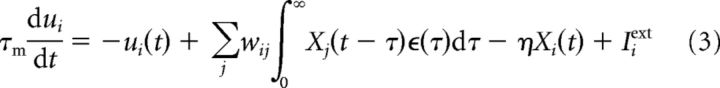
 where τ_m_ is the membrane time constant, *w_ij_* is the synaptic weight between presynaptic neuron *j* and postsynaptic neuron *i*, *I*_*i*_^ext^ is the external current (specified below), and *X_i_*(*t*) = Σ*_s_* δ(*t* − *t*_*i*_^*s*^) is the spike train of neuron *i* represented as a sum of Dirac δ-functions. The postsynaptic current kernel ϵ(τ) vanishes for τ < 0 (to preserve causality) and has the form ϵ(τ) = $\epsilon_{0}\exp\left(-\frac{\tau }{\tau_{\text{s}}}\right)$ for τ ≥ 0 with synaptic time constant τ_s_, and ϵ_0_ = τ_s_^−1^ ms mV guaranteeing normalization to ∫ϵ(*t*) d*t* = 1 mV. Afterhyperpolarization is modeled as an instantaneous current pulse with a negative sign and magnitude η ≥ 0. The instantaneous firing rate of the neuron is a nonlinear function of the membrane potential, λ(*u*), which we chose to be a linear-rectified function:

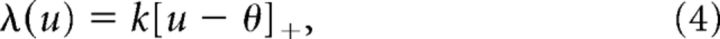
 where θ is the firing threshold, *k* is some positive constant, and the notation [*x*]_+_ denotes rectification, i.e., [*x*]_+_ = *x* if *x* ≥ 0; otherwise, [*x*]_+_ = 0. The choice of linearity for superthreshold input agrees with experimental data (Pillow et al., 2005; albeit in the retina, not in the cortex) and enables value representations consistent with the Bellman equation (see below). Deviations from linearity could also be accommodated in the model by assuming that the appropriate (inverse) nonlinearities reside downstream from the site of spike generation (e.g., in the form of short-term depression at efferent synapses; Pfister et al., 2010). The output spike train, *X_i_*, is then generated according to an inhomogeneous Poisson process with this instantaneous rate. In the following, we define values for the parameters of this network (*w_ij_*, *I*_*i*_^ext^, θ, and *k*) and show that with these choices, its dynamics solve the Bellman optimality equation (Eq. 2).

To be able to analytically study the dynamics of the network, we reduced our spiking network model to a rate model using a mean field approximation (this approximation is valid when each neuron in the network receives input from a large number of presynaptic neurons whose firing is statistically independent, or when firing rates in the network are high relative to the inverse membrane time constants of neurons). Thus, we replaced spike trains *X*(*t*) by their expected value, which are just the underlying firing rates, λ(*t*). We also replaced the convolution of the spike trains with the current kernel ∫*X*(*t* − τ) ϵ(τ) *d*τ with λ(*t*), thus ignoring delay effects, and assuming that τ_s_ is small compared with τ_m._ In our simulations, we used τ_s_ = 2 ms and 20 ≤ τ_m_ ≤ 50 ms, in line with our assumption and, importantly, also with experimentally found values for these time constants (McCormick et al., 1985). Using vector and matrix notation to conveniently collect all neurons and weights into a single equation, the dynamics of the network is as follows:

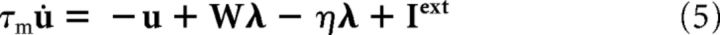


##### Neural representation of values.

We consider neurons with mixed selectivity, a phenomenon widely observed in prefrontal cortex (PFC) and important for solving complex cognitive tasks (Rigotti et al., 2013). In particular, neurons code conjunctively for state–action pairs, i.e., cell *i* codes for its corresponding (*s_i_*, *a_i_*) tuple. (For the following here, we assume discrete states and actions, and an extremely sparse population code consisting of “grandmother” neurons, but see below for an extension of our approach to continuous state and action spaces and distributed population codes.) We interpret activities in the network such that, at any point in time throughout the dynamics of the system, the sum of the firing rates of cells that code for the same state, *s*, but different actions, *a*, represents an approximation to the optimal value of *s*, *Ṽ*(*s*):

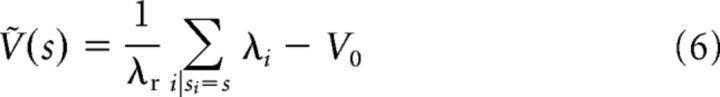
 where λ_r_ is a positive constant, to be specified in the following, and firing rates represent approximate values relative to a common baseline *V*_0_, such that *Ṽ*(*s*) > −*V*_0_ ∀*s*, which ensures that non-negative firing rates can represent all values in the task, even if some of those values are negative.

Although, as we show below, within a group of cells representing the same state *s*, asymptotically only one neuron contributes to the sum in Equation 6 (the one encoding the optimal action in that state), in general there are going to be multiple active neurons in each state contributing to this sum while activities in the network are still evolving. Indeed, the values represented by the network (Eq. 6) are approximate in the sense that initially (during early parts of the dynamics) there might not exist a policy for which *Ṽ*(*s*) is a solution to the Bellman equation (Eq. 1), and even if there is such a policy, it need not be the optimal policy. However, we will show below that on convergence of the neural dynamics, *Ṽ* not only comes to represent a valid value function consistent with the Bellman equation (Eq. 1), but it specifically represents the optimal value function corresponding to the optimal policy, *Ṽ* → V* (Eq. 2).

##### Relation to other dynamic programming algorithms.

**Figure 1.**
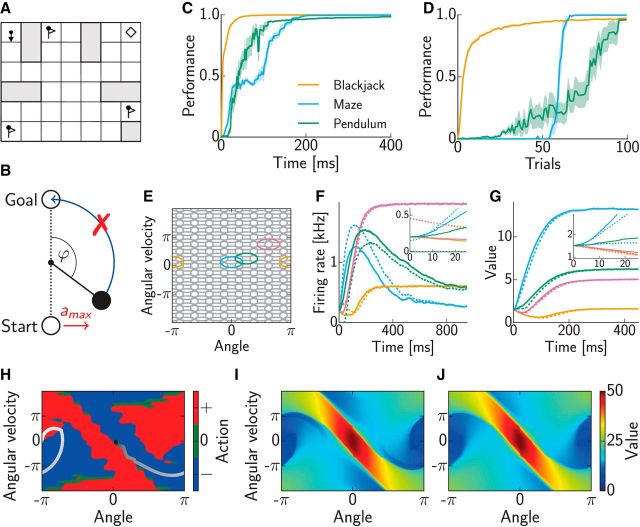
Reinforcement learning benchmark tasks. ***A***, Maze task (see Materials and Methods for details). ***B***, Pendulum swing-up task (see Materials and Methods for details). ***C***, Convergence of the dynamics toward an optimal policy representation with weights set according to the true environment. Values were computed based on spike counts up to the time indicated on the horizontal axis. Performance shows discounted average (±SEM) cumulative reward obtained by the policy based on these values, normalized such that random action selection corresponds to 0 and the optimal policy corresponds to 1. ***D***, Learning the environmental model through synaptic plasticity. In each trial, first several randomly chosen state–action pairs were experienced and weights in the network were updated accordingly, then the dynamics of the network evolved for 1 s and its performance was measured as in ***C***. ***E***, Distributed representation of the continuous state space in the Pendulum task. Ellipses show 3 SD covariances of the Gaussian basis functions of individual neurons (for better visualization only, every second basis is shown along each axis). ***F***, Activity of four representative neurons during planning. Color identifies the neurons' state-space basis functions as in ***E***, and line style shows two different initial conditions (see inset for better magnification). ***G***, Values of the preferred states of the neurons shown in ***F*** as represented by the network over the course of its dynamics. Although both initial state values (inset) and steady-state values coincide in the two examples shown (solid vs dashed lines), the interim dynamics differ because of different neural initial conditions (*F*, inset). ***H***, Policy (colored areas) and state space trajectory (gray scale circles, temporally ordered from white to black) for pendulum swing-up with preset weights. ***I***, Values actually realized by the network. ***J***, True optimal values for the Pendulum task.

Although the dynamics of our network are reminiscent of standard algorithms for computing (optimal) values, in that they are also iterative in nature and may involve the “backpropagation” of value information from successor states (Sutton and Barto, 1998), there are fundamental differences between those algorithms and ours. In particular, standard algorithms prescribe a direct interaction between values of different states (the outcome of the sum in Eq. 6), whereas in our network the interactions take place between neurons (the individual terms of the sum in Eq. 6). As a consequence, it is possible to initialize our network at two different points in the state space of its neural activities that correspond to representing the same (approximate) value function, such that as network activities evolve from these initial neural activity states they map to different trajectories in value space (Fig. 1*F*,*G*). This is clearly impossible with any algorithm operating directly on values. Thus, our network is not isomorphic to any existing algorithm and represents a novel algorithmic solution to dynamic programming.

Two classic algorithms that could be seen as being related to ours are policy iteration and value iteration. Policy iteration alternates between policy evaluation, i.e., keeping π(*a*|*s*) fixed and iterating Equation 1 until convergence to update all values, and policy improvement, changing π(*a*|*s*) such that actions more probably leading to high value states are prioritized. Value iteration performs implicit policy improvement whenever one state value is updated, *V*(*s*) ← max*_a_ r̄*(*s*, *a*) + γΣ*_s′_ P*(*s′*|*s*, *a*)V(*s′*). The closest analogy to these standard algorithms within our framework would be to select actions proportionally to the activity, π(*a_i_*|*s_i_*)=λ*_i_*/Σ_*j*|*s_j_*=*s_i_*_λ*_j_*) = λ*_i_*/*Ṽ*(*s_i_*) (ignoring constants λ_r_ and *V*_0_ for simplicity). Up to constants, λ*_i_* then equals the product π(*a_i_*|*s_i_*) *Ṽ*(*s_i_*) and is a compound representation of value function and policy. Using discretized time with step size *h*, our network dynamics update this representation according to π(*a*|*s*) *Ṽ*(*s*) ← [π(*a*, *s*) *Ṽ*(*s*) + *h*(*Q̃*(*s*, *a*) − *Ṽ*(*s*))]_+_, with state–action value *Q̃*(*s*, *a*) := *r̄*(*s*, *a*) + γΣ*_s′_ P*(*s′*|*s*, *a*)*Ṽ*(*s′*). The term in brackets compares the estimated action value *Q̃*(*s*, *a*) for action *a* with the current estimate of the state value *Ṽ*(*s*). Actions that are better than this estimate get enhanced; worse actions get suppressed and finally removed from the policy. At convergence, *Ṽ*(*s*) = max*_a_Q̃*(*s*, *a*) and π(*a*|*s*) = 1 for the optimal action and zero otherwise. In contrast to policy iteration but similarly to value iteration, our algorithm does not separate between value and policy updates, but updates both together. However, whereas value iteration updates the policy by full maximization, our algorithm performs only a small step to increase values.

##### Setting the parameters of the network.

Our design intuition is to interpret excitatory synaptic efficacies as (scaled versions of) transition probabilities and to augment the network by mutual lateral inhibition to implement the nonlinear max operation in the Bellman optimality equation (Eq. 2). Thus, the strength of the “effective” synapse connecting presynaptic neuron *j* to postsynaptic neuron *i*, *w_ij_*, summarizing the effect of asymmetric, monosynaptic excitation, *w*_*ij*_^exc^, and symmetric, bisynaptic inhibition between the two neurons, *w*_*ij*_^inh^ (mediated by inhibitory interneurons that we do not explicitly model), is as follows:

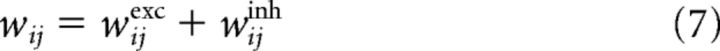
 with

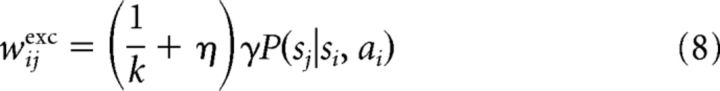


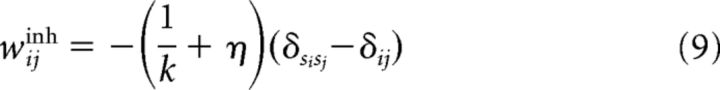
 where *k* is the slope of the firing rate nonlinearity of the neuron (Eq. 4), η is the magnitude of afterhyperpolarization (Eq. 3), γ is the temporal discounting factor (Eqs. 1 and 2), and δ is the Kronecker delta function, which equals 1 when its two indices are equal and is zero otherwise. In short, *w*_*ij*_^exc^ reflects the action of excitatory interactions encoding transition probabilities. Note that state transitions are thus encoded in the reverse direction, as a transition from state *s_i_* to *s_j_* is captured by an excitatory connection from neuron *j* to *i*. The second term, *w*_*ij*_^inh^, introduces mutual inhibition between neurons coding for the same state but different actions.

Each neuron in the network also receives external input *I*_*i*_^ext^, which is modeled as a synaptic input produced by a spike train with rate λ_r_ through a synapse with the same postsynaptic time course, ϵ(*t*), as that used for recurrent interactions (Eq. 3) and strength encoding the expected immediate reward available for the corresponding state–action pair:

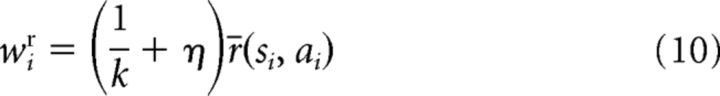
 Finally, the firing threshold represents the (appropriately scaled) magnitude of the baseline value in the following task:

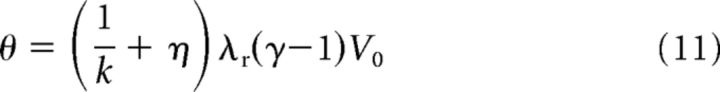
 As we show below, specifying the parameters of the network in this way ensures that it computes the optimal values for any setting of the remaining parameters (*k*, η, and λ_r_). In addition, we will also show later that the excitatory synaptic weights in the network, *w*_*ij*_^exc^ and *w*_*i*_^r^, do not need to be set “by hand” but can be learned through experience by local plasticity rules.

##### Convergence to optimal values.

Having specified all parameters of the neural network (Eqs. 4, 5, 7–10) and the mapping from neural activities in the network to approximate values of states in the task (Eq. 6), we now show that in the steady state, neural activities in the network come to represent the optimal values that satisfy the Bellman optimality equation. (Although the existence of a fixed point by itself does not generally imply that it would be ever discovered by the dynamics of a system, in this case the dynamics of our network does provably converge to this fixed point for γ < 1, i.e., this fixed point is, in fact, globally stable. However, for brevity, we omit this rather technical proof here.)

Considering the rate equations (Eq. 5), the steady state is determined by the following: ***u* = *W*λ − ηλ + *I*^ext^**. Plugging in Equations 7–10 yields the following:

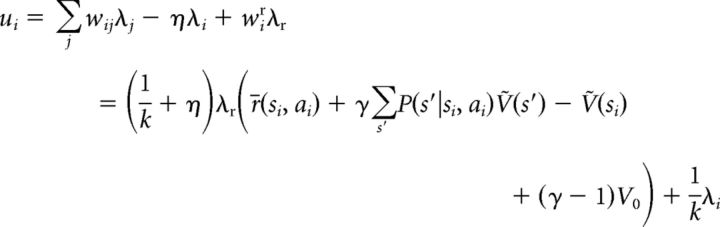
 for each neuron *i*, where we used the definition of *Ṽ*(*s*) as given by Equation 6. Since λ*_i_* = *k*[*u_i_* − θ]_+_ ≥ *k*(*u_i_* − θ), the following inequality:

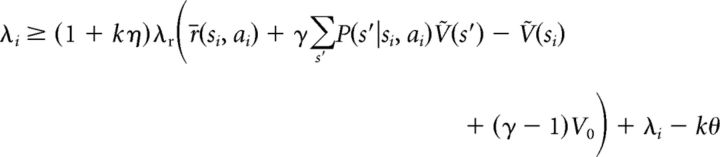
 holds for all *i*, with exact equality for positive λ*_i_*, because in this case λ*_i_* = *k* (*u_i_* − θ). Therefore, after substituting Equation 11 and canceling terms, we obtain the following:

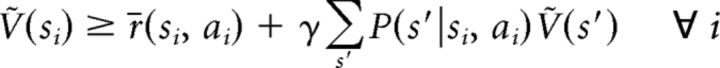
 and since all states *s* are represented by some neurons in the network, the index *i* can be dropped from the above inequality, which thus holds for all states and actions in the task:

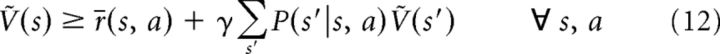
 Since *Ṽ*(*s*) = $\frac{1}{\lambda_{\text{r}}}\sum_{i|s_{i}=s}\lambda_{i}-V_{0}$ > − *V*_0_ and $\frac{1}{\lambda_{\text{r}}}>0$, there exists (at least) one strictly positive term in the sum, say for action a_*_, and therefore λ_*_ > 0. For this action a_*_, because λ_*_ is positive, Equation 12 holds with the following equality:

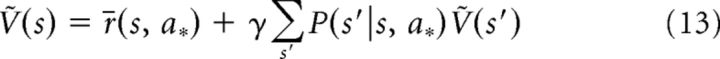
 Since Equation 12 holds for all actions, we have the following:

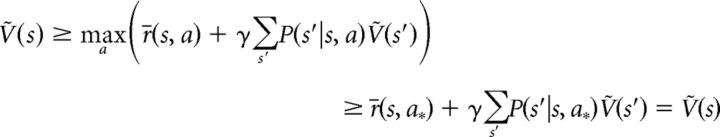
 In the last inequality, we made use of the simple fact that the maximum of a function is greater or equal to the function evaluated at any point, choosing a_*_ from above as the evaluation point.

Therefore, the last two inequalities hold with equality, and we obtain the following equality:

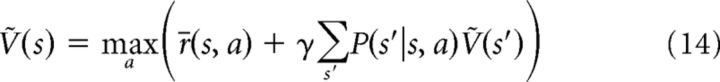
 This is exactly Bellman's optimality equation (Eq. 2), of which the solution is unique, and so in steady state, the approximate values represented by the network must be the optimal values *Ṽ*(*s*) = V*(*s*).

**Figure 2.**
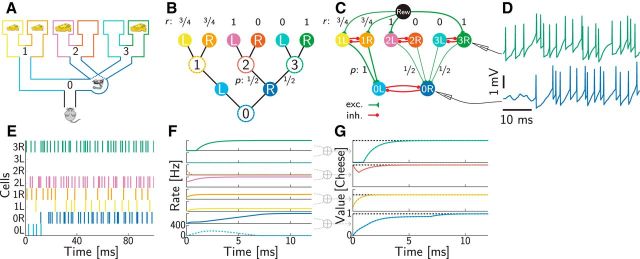
Two-step example task. ***A***, The rat moving through the maze can choose the left (L) or right (R) arm at four decision points (states 0, 1, 2, and 3). Turning right in the first step (state 0) leads to a place where one of two doors opens randomly, indicated by the coin flip. The sizes of the cheeses indicate reward magnitudes (see also ***B***). ***B***, The decision graph corresponding to the task in ***A*** is a tree for this task. Numerical values indicate rewards (r) and transition probabilities (p) for nondeterministic actions. ***C***, The corresponding neural network: action nodes in ***B*** are identified with neurons (colors). Lines indicate synaptic connections, with thickness and size scaled according to their strength. A constant external input (black) signals immediate reward. Synaptic efficacies are proportional to the transition probabilities or the (expected) reward. ***D***, Voltage traces for two neurons in ***C***. ***E***, Spike trains of all neurons. The color code is the same as in ***C***. ***F***, Activity for rate neurons with random initial values. The color code is the same as in ***C***. The line style indicates neurons coding for optimal (solid) and suboptimal (dashed) actions. ***G***, The approximate values *Ṽ*, represented by the sum of the rates in ***F***, converge to the optimal values (black dashed lines). Values of states 0–3 are shown from the bottom to top. The color code is the same as in ***B***.

Note that whereas optimal values are always unique, optimal actions in general need not be. If the optimal action in a state is unique, then only one of the terms in the sum defining the approximate value of that state, as represented by the network (Eq. 6), is nonzero and equal to λ_r_(V*(*s*) + *V*_0_). However, if multiple actions are optimal, the corresponding λ*_i_* can all be different and nonzero as long as they sum to λ_r_(V*(*s*) + *V*_0_). In this case, there is a linear subspace where **u̇** = 0, instead there is a unique fixed point. Although, in this case, the asymptotic value of **u** depends on the initial condition, the asymptotically represented value *Ṽ* does not (see Fig. 2*F*,*G*). In either case, to achieve optimal performance, one can select the action, *a_i_*, encoded by any of those neurons that are active and correspond to the current state, *s*, i.e., for which *s_i_* = *s* and λ*_i_* > 0; for example, the one with the highest activity among these is *i* = argmax_*j*|*sj* = *s*_λ*_j_*.

##### Function approximation and distributed representations.

In the foregoing, our derivations relied on discrete states, whereas real-world problems, such as navigation, often involve continuous states. Conversely, cortical population codes are distributed, rather than using the kind of extremely sparse grandmother cell-type representations assumed above. Applying a function approximation-based approach (Sutton and Barto, 1998), we consider here the important generalization to high-dimensional problems or continuous states (and actions) that resolves both these issues by letting each neuron represent a continuum of states and actions to varying degrees (as would be given by a tuning curve). In the generalized setting, each neuron *i* represents a (non-negative) basis function in the joint space of states and actions, ψ*_i_*(*s*, *a*), and so the approximate values represented by the whole network are as follows:

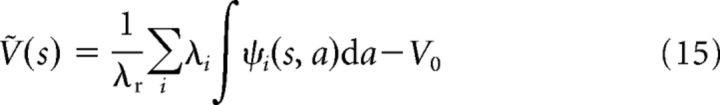
 (Note that for discrete states and actions, we can set ψ*_i_*(*s*, *a*) = δ*_s_i_s_* δ*_a_i_a_* and replace the integral by a sum, which yields Eq. 6 as a special case of Eq. 15.) Although we use linear function approximation, this only implies linearity in the bases and not in the states or actions, as the basis functions themselves may be nonlinear functions of state and action. Our approach is general in that we do not need to make explicit assumptions about the precise shape of the basis functions and only need to assume that their overlaps are positive: *K_ij_* = 〈ψ*_i_*(*s*, *a*), ψ*_j_*(*s*, *a*)〉_*L*^2^_ = ∫ψ*_i_*(*s*, *a*)ψ*_j_*(*s*, *a*)d*s* d*a* > 0 ∀ *i*, *j*.

In this case, we set the synaptic weights in the network as follows:

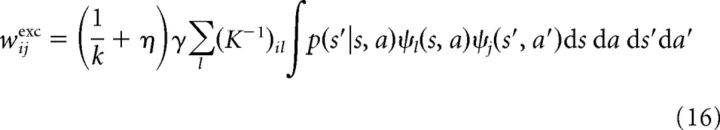


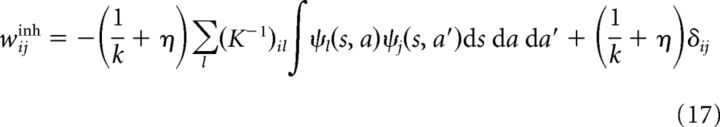


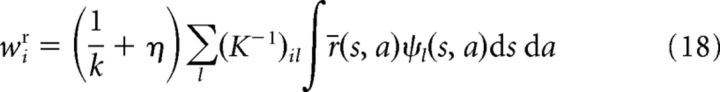
 (For discrete states and actions, we can set ψ*_i_*(*s*, *a*) = δ*_s_i_s_* δ*_a_i_a_* and replace the integrals by sums that yield *K_ij_* = δ*_ij_* and Eqs. 8–10 as a special case of Eqs. 16–18.)

The firing threshold again represents the (appropriately scaled) magnitude of the baseline value in the task (compare Eq. 11):

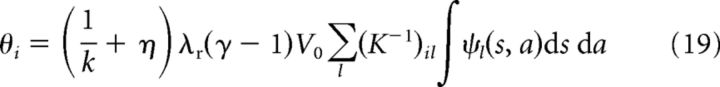
 In analogy to the derivations above establishing the equivalence of *Ṽ* to V* at steady state, we can derive the following. Plugging in Equations 16–18 into the steady-state equation yields the following:

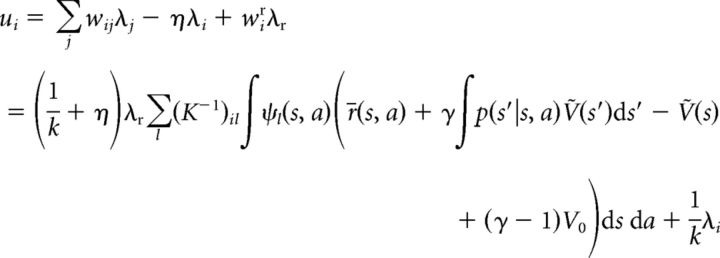
 for each neuron *i*, where we have used the definition of *Ṽ*(*s*) as given by Equation 15. Since λ*_i_* = *k*[*u_i_* − θ*_i_*]_+_ > *k*(*u_i_* − θ*_i_*), the following inequality:

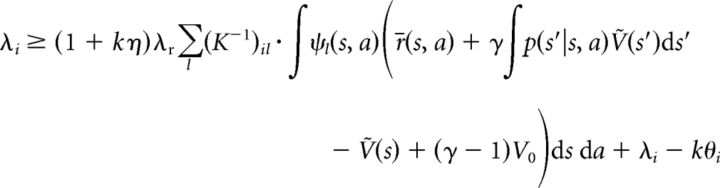
 holds for all *i*, with exact equality for positive λ*_i_*, because, in this case, λ*_i_* = *k* (*u_i_* − θ*_i_*). Therefore, after substituting Equation 19 and canceling terms, we obtain the following:

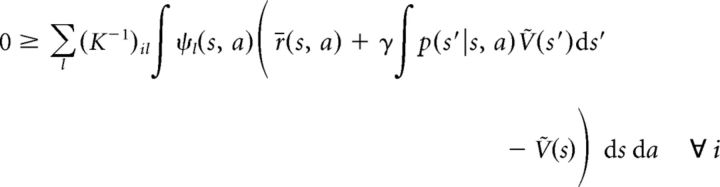
 By definition, *K* is a non-negative matrix, and left-multiplication with it yields the following:

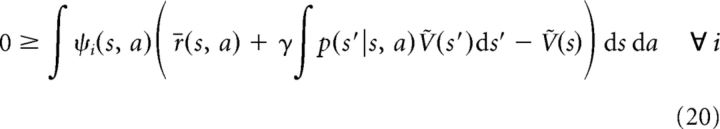

Equation 20 expresses the fact that instead of the exact match between the value function represented by the network, *Ṽ*(*s*), and the true optimal value function, V*, which we had in the discrete case (Eq. 14), here we have an approximate match between the two. This approximation is such that the integral of *Ṽ*(*s*) and V* in the neighborhood (defined by ψ) of only a finite (but potentially large) number of “control points” in the joint state–action space is being matched, where the neurons of the network represent these control points. Also in analogy to the discrete case, an action for each state *s* can be selected by maximizing the predicted value according to the network's representation: a* = argmax*_a_*Σ*_i_*λ*_i_*ψ*_i_*(*s*, *a*). However, because of the approximate nature of this representation, there is no guarantee that the optimal policy can be found. Nevertheless, in benchmark tests, we found that a very close approximation of it was still achieved (see below; Fig. 1*I*,*J*).

##### Learning.

For a known transition and reward model, the weights can be set to their values as described in the previous sections, whereas if the model is unknown (as it is initially in biological settings), they need to be learned through interaction with the environment. Here we show that in our model, a local plasticity mechanism enables learning the correct weights.

We obtain the corresponding plasticity rule by minimizing the squared error between the current weight and the target weight, i.e., the right-hand side in Equation 8 or 10 [note that inhibitory weights do not depend on the environment (Eq. 9) and are hence assumed to be set from the beginning]. This yields a delta rule of the form Δ*w* = α(*t* − *w*) with target *t* and learning rate α. (This formulates plasticity as consisting of abrupt weight changes Δ*w* at certain time points. An online continuous time scenario *ẇ* = Σ*_t′_ F*(*t* − *t′*)Δ*w*(*t′*) with decision times *t′* and some function *F*(*t*), e.g., a (low-pass-filtered) square pulse as in the study by Friedrich et al. (2010) instead of a delta function, is also possible.) In our case, the target values include the unknown probabilities, which are only observed in the form of samples. If in state *s* action *a* is performed yielding reward *r* and leading to state *s′*, the online estimation rules for updating after each transition yield the following postsynaptically gated form of plasticity:

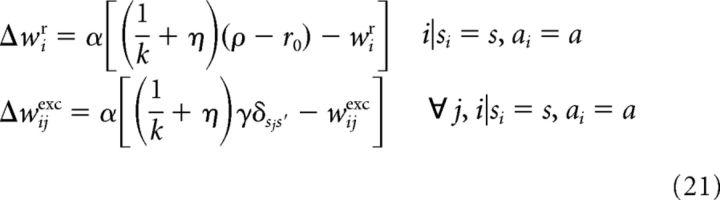
 where ρ = *r* + *r*_0_ is the rate of the external input (the same biological quantity as that described by λ_r_, but denoted differently to emphasize the different functional role it plays during learning than during the computation of the value function), with *r* the currently obtained reward and *r*_0_ a sufficiently large positive constant such that ρ always remains non-negative. Thus, during learning, the activities of neurons in the network and the rates of the external input to the network are determined by the immediate experience of the animal, rather than the internal dynamics of the network as described in the previous sections. Specifically, performing a state–action transition activates the corresponding neurons in the network (Mushiake et al., 2006) and sets the rate of the external input, making this information available to the synapse in the form of presynaptic and postsynaptic activities. Beside this, plasticity also involves simple synaptic scaling. Hence, all quantities are locally available at the corresponding synapse.

In the case of function approximation, the update rules performing (stochastic) gradient steps on the quadratic error between the current weights and the optimal weights defined in Equations 16–18 are as follows:

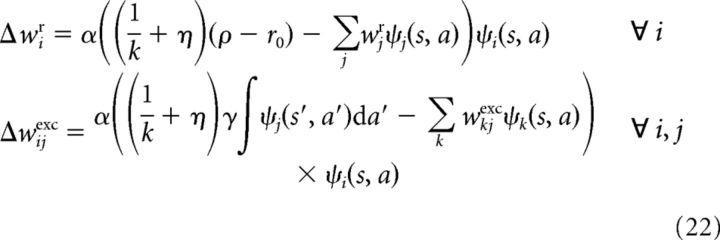
 For ψ*_i_*(*s*, *a*) = δ*_s_i_s_* δ*_a_i_a_*, Equation 22 reduces to Equation 21. In contrast to the discrete case, here synaptic modification, at least its synaptic scaling component, becomes more global in that it also depends on neighboring efferent synapses of the presynaptic neuron. This challenges the locality of the plasticity rule, but it may be possible to effectively approximate this term by intrinsic plasticity (Savin et al., 2014), thus preserving locality.

##### Validation on benchmark tasks.

To validate our network, we tested it on three well known challenging sequential decision-making benchmark tasks from the reinforcement learning literature.

The first, “Blackjack,” was based on a scenario described by Sutton and Barto (1998): the agent is the player in a game of blackjack (played against the dealer). Standard rules of blackjack hold but are modified to allow the player to (mistakenly) hit on 21. States jointly encode the hand value of cards the player is holding, the value of the open card of the dealer, and whether the player holds a usable ace. The two available actions are to draw or to stop. We used a reward of 0 for a loss, ½ for a draw, and 1 for a win.

Second, in the “Maze” task (Dearden et al., 1998; Strens, 2000), the agent needs to navigate a “grid-world” maze by moving left, right, up, or down by one square at a time (Fig. 1*A*). If it attempts to move into a wall, its action has no effect. The task is to move from the start (top left) to the goal (top right) collecting the flags on the way. When the agent reaches the goal, it receives a reward equal to the number of flags collected. On top of this, a γ < 1 discounting factor encourages the agent to complete the task in as few moves as possible. The problem is made more challenging by assuming that the agent occasionally “slips” and moves in a direction perpendicular to that intended (with probability 0.1).

Third, the “Pendulum swing-up” is a problem of swinging up a rigid pendulum suspended by a 1 df joint from its base, by applying torque to rotate it (Fig. 1*B*; following Deisenroth et al., 2009). The dynamics of this system are described by the equation ϕ̈(*t*) = $\frac{-\mu \dot{\phi }\left(t\right)+mgl\sin\left(\phi \left(t\right)\right)+a\left(t\right)}{ml^{2}}$, where −π ≤ ϕ ≤ π is the angle of the pendulum (ϕ = 0 is vertical upward), μ = 0.05 kg m^2^/s is the coefficient of friction, *l* = 1 m is the length of the pendulum, *m* = 1 kg is the mass of the pendulum, *g* = 9.81 m/s^2^ is the gravitational constant, and *a*(*t*) is the torque applied at time *t*. States encode angular position and speed, ***s*** = (ϕ, ϕ̇), and the initial state is the stable equilibrium of the upside-down hanging pendulum at rest (π, 0). The task is to swing the pendulum up and to balance it in the inverted position around the goal state, (0, 0). The immediate reward function is a Gaussian centered on the goal state: *r*(***s***) = exp(−***s*** diag(1, 0.2) ***s****^T^*). This task is challenging because there are only three discrete actions available, –, 0, and +, corresponding to an applied torque of *a* = −5, 0, and 5 Nm, respectively, which is not sufficient for a direct swing-up.

For the first two tasks, we used a nondistributed (“look-up table”) representation, with each neuron coding for a different state–action pair. For the pendulum swing-up, we used neurons with Gaussian receptive fields on a grid to cover the phase space and matched their width σ to the grid spacing Δ*_s_* via σ = ½Δ_s_ (Fig. 1*E*). As grid spacing, we chose Δ*_s_* = $\frac{\pi }{8}$ rad for angular position ϕ and Δ*_s_* = $\frac{\pi }{8}$ rad/s for angular velocity ϕ̇, respectively, yielding 16 × 33 grid points and a total of 16 × 33 × 3 = 1584 neurons. For Blackjack, which is a finite horizon task, we used discount factor γ = 1, whereas for the infinite horizon tasks (Maze and Pendulum) we used γ = 0.98. Other parameters were *k* = 1 Hz/mV, η = 20 mV, τ_m_ = 20 ms, and τ_s_ = 2 ms in all simulations. The reward unit fired with a constant rate of λ_r_ = 400 Hz. The firing rate of all neurons was initialized at 0 Hz, except for that shown in Figure 1, *F* and *G*, where we tested two different initial conditions: one corresponding to all neurons firing at 200 Hz and one corresponding to neurons encoding action –, 0, and + firing at 0, 150, and 450 Hz, respectively. Hence, the sum over neurons with the same preferred state but different encoded actions was 600 Hz in both cases, which consequently meant (attributable to Eq. 15) that the approximate value functions represented by these two different initial conditions were precisely identical. We used a small fixed learning rate α = 0.01 for Blackjack and α = 0.05 for Pendulum and Maze.

We first simulated the tasks with weights set according to perfect knowledge of the task structure, i.e., the set of action–outcome relationships and associated expected immediate rewards (Eqs. 8–10 for discrete, Blackjack, and Maze and Eqs. 16–18 for continuous state spaces and Pendulum). As the measure of performance, we used the normalized discounted cumulative reward, such that random action selection corresponded to performance 0 and following the optimal policy corresponded to 1. We ran the dynamics for some time, *t*, and calculated the policy, and the expected return under the inferred policy, based on the activities recorded so far, in the time interval [0, *t*]. Note that with spiking neurons, time plays a twofold rule: it allows the dynamics of the network (Eq. 3) to converge, but it is also required to be able to estimate firing rates (and thus the corresponding value function; Eq. 6) from spike counts. (In fact, one could allow an initial “burn-in” time and disregard the activity during the burn-in period for the value computation, trading off waiting time and the accuracy of the obtained policy, but we chose the simpler solution of having no burn-in period.) In general, convergence time depended on task difficulty, but in all three tasks tested, a nearly optimal policy has been already found after a mere 200 ms (Fig. 1*C*). In the Maze task, rather sudden increases in performance occurred as with increasing planning time the policy was to pick up one flag first, then two, and finally all three flags.

For the Pendulum task, the firing rate of a subset of neurons, obtained by filtering their spike trains with an exponential moving average (τ = 40 ms), is shown in Figure 1*F* for two different initial conditions of neural activities that correspond to representing the same (approximate) value function (see above). The values represented by the network vary as the dynamics evolve and coincide again on convergence as the network comes to represent the unique optimal value function (Fig. 1*G*). However, note that despite identical value functions represented in the initial and steady states of the network, the value functions represented while network activities are still evolving are different for these two trajectories, which would be impossible if the dynamics operated directly in value space. The convergence time of neural activities differed and could be particularly slow for neurons with basis functions in a region of state space where competing actions led to similar returns, as a near-optimal policy was established before the last neuron's activity converged (Fig. 1*C*). The inferred trajectory after letting the network dynamics evolve for 1 s is depicted in Figure 1*H*, together with the policy for the relevant section of phase space. Comparing Figure 1, *I* and *J*, reveals that the actually obtained cumulative reward closely matches the true optimal value.

Figure 1*D* shows that the weights can also be successfully learned through interaction with the environment according to Equation 21 or 22, for discrete or continuous state space problems, respectively. To focus on the ability to exploit knowledge extracted about an environment, we simplified exploration using a form of parallel sampling (Kearns and Singh, 1999), which samples at each iteration for every state–action pair a random next state. Because parallel sampling is not directly applicable to continuous state spaces, as there is an infinite number of states, we adapted it accordingly and sampled in each trial uniformly randomly 500 states from the phase space region depicted in Figure 1, *E* and *H–J*, and for each state chose each of the three actions in turn (resulting in a total of 1500 state–action pairs available for learning in each trial). After learning, in each trial, the dynamics of the network were run for 1 s, sufficiently long to ensure convergence, so that suboptimal performance reflected a mismatch between true transition and reward probabilities in the environment and their model embedded in the synaptic weights of the network. In the maze task, a rather abrupt jump in performance happened as the agent learned to reach the goal location. In the individual runs of the pendulum task, a sudden increase in performance occurred as soon as the pendulum was swung up for the first time. Such changes occurred after different numbers of trials, resulting in the average curves shown with gradual increase and large confidence regions.

##### Planning-as-inference.

Planning-as-inference has emerged recently as a powerful new way of solving MDPs in a machine learning context (Toussaint and Storkey, 2006) and also as a theory of how cortical networks may perform model-based sequential decision making (Botvinick and Toussaint, 2012). Thus, we implemented it so that we could contrast it with our network directly in a number of scenarios, in terms of their computational performance as well as their ability to account for relevant biological data.

Planning-as-inference treats the task of planning in an MDP as a special case of probabilistic inference over a set of random variables, *X*, that include the states (*s_t_*), actions (*a_t_*), and immediate rewards (*r_t_*) in future time steps (*t*), the total cumulative reward (*R*), and the policy (π*_t_*) that the decision-making agent might choose to adopt for selecting actions (which may also be time dependent). The agent then represents the future as a joint probability distribution over *X*, *P*(*X*) [cf. Solway and Botvinick (2012), their Fig. 2*D*]. For planning, the agent uses this joint distribution such that it conditions on the cumulative reward, *R*, starting from the premise that its actions will yield high rewards, and then uses inverse (Bayesian) inference to discover the policy that renders this assumption most plausible, i.e., to compute *P*(π*_t_*|*R*).

To relate planning-as-inference to neural data, Solway and Botvinick (2012) used belief propagation (BP) for inferring the policy. As a first step for inference by BP, the joint distribution of the variables of interest, *P*(*X*), is expressed as a product of factors: *P*(*X*)∝ ∏*_f_* φ*_f_*(*X_f_*), where each factor *f* expresses probabilistic dependencies, φ*_f_*, between a subset of the variables, *X_f_*. For planning-as-inference, there are four kinds of factors that are relevant: those expressing transition probabilities, *P*(*s*_*t*+*1*_|*s_t_*, *a_t_*); those expressing reward probabilities, *P*(*r_t_*|*s_t_*); those expressing action selection probabilities, *P*(*a_t_*|*s_t_*, π*_t_*); and those that express the way all the immediate rewards make up the total cumulative reward, *P*(*R*|{*r_t_*}). Note that each factor includes several variables and that the same variable is generally included in several factors. Inference by BP then proceeds by recursively passing “messages” from variables to factors, μ_*x*→*f*_(χ) = ∏_*g*∈*F*_*x*_^−*f*^ ν_*g*→*x*_(χ)_, and from factors to variables, ν_*f*→*x*_(χ) = ∑_*Y* = {*X*_*f*_^−*x*^, *x* = χ}_φ*_f_*(*Y*)∏_*y*∈*X*_*f*_^−*x*^ = γ_ μ_*y*→*f*_(γ), and the marginal of any variable *x* can be obtained as a product of all the messages including it: *P*(*x* = χ) = ∏_*f*∈*F_x_*_ν_*f*→*x*_(χ), where *F_x_* and *F*_*x*_^−*f*^ denote all the factors including variable *x* (except *f* for *F*_*x*_^−*f*^); and *X_f_* and *X*_*f*_^−*x*^ denote all the variables included in factor *f* (except *x* for *X*_*f*_^−*x*^). To allow the diffusion of information across temporally distributed events and to arrive at a deterministic policy, following Botvinick and An (2009), we ran BP several times, such that on each iteration, the inference of the posterior policy used the posterior from the previous iteration as the new prior.

Following Rao (2004) and Solway and Botvinick (2012), we mapped (loopy) BP to a neural network in which neural activities represented the messages, and connections between the neurons embodied the interdependencies of the messages such that only local interactions were necessary between neurons (although the effect of multiple presynaptic cells on the same postsynaptic cell, for factors including several variables, needed to be multiplicative). In particular, Solway and Botvinick (2012) corresponded the message from factor *P*(*a_t_*|*s_t_*, π*_t_*) to the variable representing the optimal action in the current state, *a*_t_^*^, to (normalized) neural activities recorded in lateral intraparietal area, which change approximately monotonically in experiments. However, the data we sought to account for, recorded in pre-SMA (Sohn and Lee, 2007), showed markedly nonmonotonic activity time courses (see Fig. 6*A*). Therefore, to obtain model time courses directly comparable with neural data, we scaled the magnitude of the message by a nonmonotonic Gaussian envelope (see below at simulation details).

Using BP for planning-as-inference also suffers from a computational drawback. As the messages are defined recursively, BP is only guaranteed to converge when there are no circular dependencies between them. Unfortunately, in the case of planning-as-inference, this is not the case in general, and so loopy BP needs to be performed without such guarantees (Koller and Friedman, 2009). Indeed, we verified in simulations that this approach happens to fail (data not shown) on our specific illustrative example task requiring closed-loop control (Fig. 2).

Using more advanced inference methods, e.g., the junction tree algorithm (Lauritzen and Spiegelhalter, 1988), instead of loopy BP, for planning-as-inference is guaranteed to result in the optimal policy even in situations requiring closed-loop control. However, for situations in which open-loop control suffices, such as the experimental task modeled in Figure 6, this will result in neural time courses that are identical to those obtained with loopy BP (see Fig. 6) and are, therefore, equally at odds with experimental data (see above). Moreover, in contrast to loopy BP, the junction tree algorithm lacks even more the plausibility of being performed in the brain. For these reasons, we do not report here separate results obtained with the junction tree algorithm.

##### Simulation details.

For the two-step example task (Fig. 2), we used the same parameters as in the benchmark tasks, i.e., *k* = 1 Hz/mV, η = 20 mV, τ_m_ = 20 ms, τ_s_ = 2 ms, and λ_r_ = 400 Hz.

**Figure 3.**
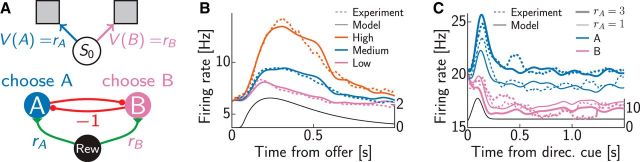
Time course of neural activity in a binary choice task. ***A***, The task (top) consisting of a single state (*s*_0_) and two actions (A and B) associated with different values (which, in this case, were also their immediate rewards, *r*_A_ and *r*_B_) and the corresponding neural network (bottom). ***B***, Average population activity for offer value cells (dashed line) from the study by Padoa-Schioppa (2013) and simulation results (solid line). Trials were divided into three groups depending on the offer value (colors). ***C***, Average population activity (dashed line) from the study by Roesch and Olson (2003) and model results (solid line). Trials were divided depending on whether the cell encoded the optimal action (blue) or not (purple) and on whether the reward was large (thick) or small (thin). The activity of the reward input used in the simulations is shown as a black curve in ***B*** and ***C*** with the corresponding *y*-axis plotted on the right side.

**Figure 4.**
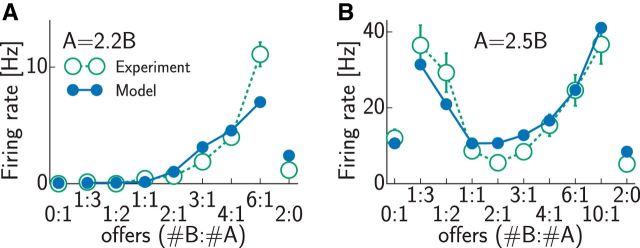
Value dependence of neural firing rates in a binary choice task in experiments (open green circles; adapted from Padoa-Schioppa and Assad, 2006) and simulations (filled blue circles). ***A***, Neuron encoding offer value of option B. One unit of juice A was worth 2.2 units of juice B. ***B***, Neuron encoding chosen value, 1A = 2.5B. Error bars show SEM and were often smaller than the symbols.

**Figure 5.**
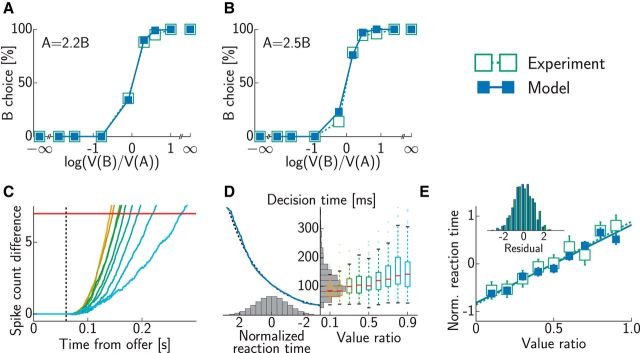
Psychometric and chronometric curves in a binary decision-making task. ***A***, ***B***, Choice probabilities in experiments (open green squares; Padoa-Schioppa and Assad, 2006) and simulations (filled blue squares) for two different relative values of the two juices: 1A = 2.2B (***A***) and 1A = 2.5B (***B***). ***C***, Difference between the cumulative spike counts of populations representing the two potential choices in the model. Accumulation starts with sensory delay (dashed line; compare input onset in Fig. 3*B*). When a threshold (red line) is reached, a decision is made. Colors indicate different value ratios as in ***D***. ***D***, Decision time distributions in the model. Right, Dependence of raw decision times on the value ratio (colored Tukey's boxplots) and their overall distribution across all value ratios (gray histogram). Left, Normalizing function (solid blue line), together with a logarithmic fit (dashed black line), which transforms the raw decision time distribution into a standard normal distribution (gray histogram). ***E***, Normalized reaction times (±SEM) as a function of value ratio in experiments (open green squares; Padoa-Schioppa and Assad, 2006) and simulations (filled blue squares). Lines show least squares fits (dotted green, experiments; solid blue, simulations); the inset shows distribution of residuals after fitting (green bars, experiments; blue bars, simulations).

For modeling the binary choice tasks (Figs. 3–5), the strengths of the synaptic weights carrying the reward-dependent external input were equal to the offered values measured in units of option B. Other parameters were *k* = 1 Hz/mV, η = 0 mV, τ_m_ = 25 ms, and τ_s_ = 2 ms in all simulations. For matching experimental data, we added a constant baseline firing rate to the rates computed in the model (λ, Eq. 4), without affecting the represented values. Initial and baseline firing rates (∼5.2 Hz for Fig. 3*B*, ∼16.5 Hz for Fig. 3*C*, and 0 Hz for Figs. 4 and 5) were qualitatively matched to experimental data and were the same across all conditions (pairs of offer values). Time-dependent firing rates in Figure 3 were obtained by convolving the spike trains with a Gaussian kernel (σ = 40 ms) and averaging over 30 simulation runs. The results in Figures 4 and 5 were obtained by averaging over 100 runs.

**Figure 6.**
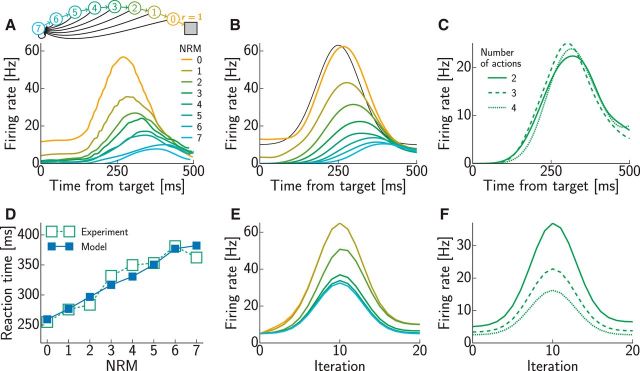
Sequential decision making. ***A***, An example neuron in pre-SMA showing activity modulated by the NRMs (colored lines; Sohn and Lee, 2007): amplitude decreases and delay increases with NRM. The inset shows task structure: colored circles indicate states (numbers show NRMs), arrows show state transitions (colored lines, correct action; black lines, incorrect action), and the gray square represents terminal state with reward (modeled as *r* = 1). ***B***, Activity time courses of an example model neuron as a function of NRMs. The color code is the same as in ***A***. The black line shows activity of the reward input chosen to fit experimental data. ***C***, Activity time courses of an example model neuron as a function of the number of available actions (1 correct, others incorrect) in the state with NRM = 3. ***D***, Experimental (open green squares; Sohn and Lee, 2007) and simulated (filled blue squares) reaction times increased approximately linearly with NRMs. Error bars (SEM) are all smaller than the symbols. ***E***, ***F***, Predictions of planning-as-inference for neural time courses as a function of NRMs (***E***) and number of available actions (***F***). The color code is the same as in ***B*** and ***C***.

**Figure 7.**
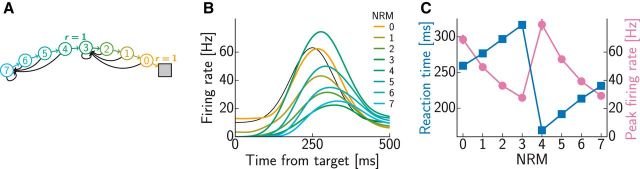
Predictions for a novel sequential decision-making task. ***A***, Task structure with rewards in two distinct steps; symbols are as in Figure 6*A* (inset). ***B***, Simulation results for the suggested task with added intermediate reward. The color code and activity of the reward input are as in Figure 6*B*. ***C***, Reaction times (blue squares) and peak firing rates (purple circles) from the simulations in ***B*** vary nonmonotonically with NRM. Error bars (SEM) are often smaller than the symbols.

**Figure 8.**
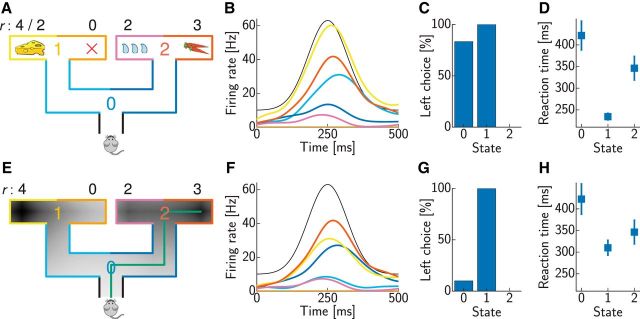
Reinforcer devaluation. ***A***, The rat moving through the maze can turn left or right at three decision points (states 0, 1, and 2; colored numbers). The numbers above the terminal positions indicate the corresponding rewards. Devaluation decreases the reward associated with cheese (top left) from a baseline level of 4 to a devalued level of 2. [Adapted from Niv et al. (2006).] ***B***, Simulated firing rates with baseline reward values. Colors indicate the state–action pair encoded by each cell, following the color scheme in ***A***. The activity of the reward input (black) is as in Figure 6*B*. ***C***, ***D***, Choice probabilities (***C***) and reaction times (***D***; ±SEM) in each state. ***E***, Activity profile for a spreading-activation model (darker means increasing activity). The path of an agent following the activity gradient (green) yields only a reward of 3 instead of the optimal 4. ***F–H***, Same as in ***B–D*** following devaluation in our model. Note the change in the choice at the initial decision point (state 0).

For modeling the sequential decision-making tasks (Figs. 6–8), the neural parameters were *k* = 1 Hz/mV, η = 3 mV, τ_m_ = 50 ms, and τ_s_ = 2 ms. The discount factor was γ = 0.7. The initial firing rates were qualitatively matched to the experimental data, separately for each step of the task [indexed by the number of remaining movements (NRMs)], and no baseline rate was used. Time-dependent firing rates were again obtained by convolving the spike trains with a Gaussian kernel (σ = 40 ms) and averaging over 30 simulation runs.

The activity profile of the reward input, λ_r_(*t*), was chosen in all cases to yield realistic neural time courses in the model. Note, however, that our focus was on the differences between these time courses between different conditions, whereas the activity profile of the reward input was identical across conditions, so this by itself could not account for our main results, which were instead characteristic of the particular intrinsic dynamics of our network. Specifically, in Figure 3*B*, we chose a double-exponential time course λ_r_(*t*) = $\frac{\lambda_{\max}}{\alpha }\left(\exp\left(-\frac{t-t_{\Delta }}{t_{\text{d}}}\right)-\exp\left(-\frac{t-t_{\Delta }}{t_{\text{r}}}\right)\right)\Theta \left(t-t_{\Delta }\right)$ with maximal firing rate λ_max_ = 2.6 Hz, rise time *t*_r_ = 110 ms, decay time *t*_d_ = 300 ms, and delay *t*_Δ_ = 60 ms, where α = ${\left(\frac{t_{\text{r}}}{t_{\text{d}}}\right)}^{-\frac{1}{1-t_{\text{d}}/t_{\text{r}}}}\left(1-\frac{t_{\text{r}}}{t_{\text{d}}}\right)$ is a normalizing constant, and Θ is the Heaviside step function, i.e., Θ(*x*) = 1 for *x* ≥ 0 and Θ(*x*) = 0 otherwise. In Figure 3*C*, we chose a raised Gaussian time course λ_r_(*t*) = λ_base_ + (λ_max_ − λ_base_) exp(− (*t* − *t*_p_)^2^/τ^2^) with baseline firing rate λ_base_ = 4.5 Hz, maximal firing rate λ_max_ = 27.9 Hz, peak time *t*_p_ = 95 ms, and duration τ = 30 ms. In Figures 4 and 5, we again chose the double-exponential time course with the same time constants and maximal firing rates that differed across panels: λ_max_ = 1.6 Hz (Fig. 4*A*), λ_max_ = 5.4 Hz (Fig. 4*B*), and λ_max_ = 70 Hz (Fig. 5). The higher maximal firing rates for behavioral data compared with neural data represent population firing rates and capture the fact that decisions are encoded by populations and not single neurons. In the sequential decision-making tasks (Figs. 6–8), we also chose a raised Gaussian time course with baseline firing rate λ_base_ = 10 Hz, maximal firing rate λ_max_ = 75 Hz, peak time *t*_p_ = 250 ms, and duration τ = 60 ms. For the behavioral data in Figures 6*D* and 7*C*, we increased the baseline and maximal firing rate by a factor 10.

For modeling the sequential decision-making task (Fig. 6) by planning-as-inference, we ran noiseless rate-based simulations, rather than simulating a spiking neural version, thus merely adding noise, and averaging over various runs to reduce the noise (Fig. 6*E*,*F*). For this, we used the code of Solway and Botvinick (2012) with loopy BP, which yields activations in the interval [0,1] that represent probabilities. To obtain activation patterns as similar to the experimental data (Fig. 6*A*) as possible, we scaled the output of the simulated activities by a time (iteration)-dependent factor 10 + 55 exp $\left(-\frac{{\left(t-10\right)}^{2}}{4^{2}}\right)$, in analogy to the Gaussian activity profile of the reward inputs we chose in our model (see above).

For modeling behavior, we assumed that a decision was made when the spike count difference between any two populations encoding different currently available actions reached a threshold θ_dec_ (Fig. 5*C*). When the threshold was reached, a decision in favor of the action encoded by the population with higher spike count was made deterministically. Thus, once the parameters of the neural simulations were determined, only one more parameter, determining the setting of the threshold, was fitted to match the overall scale of reaction times in the behavioral data. Specifically, in the binary decision task (Fig. 5), this threshold was set such that the average decision times over the offer value ratios in Figure 5*E* were similar to those reported in experiments. From Padoa-Schioppa et al. (2006), we extracted a mean reaction time of 490 ms and subtracted a nondecision time of 370 ms (Resulaj et al., 2009), resulting in a mean decision time of *t*_dec_ = 120 ms. Accumulation of spike counts started with the onset of the reward input, i.e., *t*_Δ_ = 60 ms after offer presentation (Fig. 5*C*, dashed line). This sensory delay contributed to the nondecision time; hence, we chose the threshold θ_dec_ = 7 such that it was crossed after 180 ms (*t*_Δ +_ *t*_dec_), on average. In the sequential decision-making tasks (Figs. 6–8), the threshold was discounted with the time horizon of the current decision, corresponding to NRMs (Fig. 6*A*): θ_dec_ = θ_0_γ^NRM^, where γ = 0.7 was the discount factor chosen to match neural activation time courses as described above, and θ_0_ = 70 was chosen to match the average reaction time reported in the experiment (∼320 ms; Sohn and Lee, 2007). To account for the shorter time horizon in Figure 8, we used θ_0_ = 7 for this task.

For modeling the spreading activity profile in Figure 8*E*, we first calculated for each position its distance *d* from each goal, using the ℓ_1_-l. (The ℓ_1_-l was used to capture the fact that the animal is forced to move along the center of the corridors, i.e., either horizontally or vertically, in this case.) The contribution of each goal to the steady-state activity at a given position was then computed as *r*e^−*d*/γ^, with reward size *r* (as shown in the figure) and attenuation length scale γ = 1.2 times the length of the second segment of the green path in Figure 8*E* (the exact value of γ did not affect our results). Finally, the overall activity at each position was obtained by a linear superposition of these contributions. For simplicity, we allowed activity to spread symmetrically along every corridor, e.g., even from state 2 via 0 to 1. However, restricting activity spread to be strictly retrodromic (i.e., only toward the initial position of the rat), thus breaking symmetry and preventing the rat to ever turn around, did not change our conclusions. We constrained the path along the gradient to stay in the middle of the corridors.

## Results

The central difficulty of sequential decision making is that actions need not simply be chosen based on the immediately ensuing rewards (or punishments); instead, their long-term consequences need to be considered, which in turn depend on several other actions we have already made or will make in the future. To solve this problem flexibly, an internal model of the environment needs to be maintained and used to compute these consequences before making each new decision. Although there exist well known computer algorithms that can solve this problem (collectively known as dynamic programming) and there is evidence for the behavioral and neural correlates of such computations, it is unknown how networks of spiking neurons actually implement them.

We were able to show mathematically that a recurrently coupled network of spiking neurons, when connected appropriately, is able to represent, through its internal dynamics, the value of the best action in a given situation (see Materials and Methods). This value expresses the average total future reward attainable from the current situation if the next action as well as all future actions are selected optimally, and it is a central quantity of mathematical theories of sequential decision making (and optimal control) because it itself allows the selection of optimal actions. Crucially, the value computed by our network also depends on an internal model of the environment, in particular on the knowledge of how situations follow from each other depending on the actions chosen (so-called transition probabilities), and how they result in immediate rewards (reward probabilities). In the case of a recurrent neural network, this internal model is implicitly embedded in the efficacies of the synapses connecting the neurons to each other (as well as to an external input signaling immediate rewards). Thus, we also showed how (semi-)local forms of synaptic plasticity can tune synapses in the network appropriately, in an experience-dependent manner using reward-prediction and state-prediction errors akin to those described experimentally (Gläscher et al., 2010), so that the internal model validly captures the relevant statistical structure of the environment. As the computations in our network did not directly correspond to any previously described dynamic programming algorithm, we were interested in how well it approximated optimal performance and whether there were signatures of the particular dynamics of our network in cortical areas involved in decision making.

### Illustrative example task

Besides benchmarking the performance of our network extensively on a range of standard reinforcement learning tasks of increasing complexity (see Materials and Methods; Fig. 1), we also studied in more detail its underlying neural dynamics in a simple but nontrivial artificial decision-making task (Fig. 2). We considered a two-step navigation task in a simple maze with one stochastically operating door (Fig. 2*A*). This task corresponded to a simple decision tree with four states, in each of which two alternative actions were available: turning left or right (Fig. 2*B*). Although this task is simple, it is not trivial. “Open loop” control, which precommits to one action sequence already at the start and plays it out regardless of what happens on the way, would perform action L in the first step and L or R in the second step, yielding reward ¾ compared with ½ (on average) if choosing R first and then either L or R. However, optimal behavior requires “closed loop” control to first take action R and then select the next action depending on the state that is reached through the stochastically operating door (L for 2 and R for 3), achieving a total reward of 1. Hence, this deceptively simple task differentiates between optimal open-loop and closed-loop action selection. Interestingly, the only proposal so far for how neural networks may perform model-based decision making (Solway and Botvinick, 2012) failed on the specific task considered here (data not shown; see also Materials and Methods).

In our neural network, which solved this task successfully, each action in each state was represented by a separate neuron (Fig. 2*C*, colored circles), receiving external input signaling immediate reward (Fig. 2*C*, black circle). Excitatory synaptic efficacies were proportional to the transition probabilities and the (expected) reward (Fig. 2*C*, green connections), respectively. Inhibitory synapses between neurons coding for different actions in the same state were all of the same strength (Fig. 2*C*, red connections). The dynamics of this spiking network quickly, within 10 ms, settled into a steady state (Fig. 2*D*,*E*), which was most apparent when measured in terms of firing rates (Fig. 2*F*). In this steady state, the network correctly represented the optimal values (Fig. 2*G*). This representation was achieved by eventually only one neuron remaining active in each group representing a state (because the optimal action was unique), through competition mediated by lateral inhibition within the group (Strait et al., 2014). The only exception for this was the group representing state 1, in which both actions had exactly the same value and thus both corresponding neurons remained active (Fig. 2*F*). The asymptotic firing rates of these neurons depended on the initial conditions, but the state value that was represented by their sum did not (data not shown). The transient behavior of the network was also revealing. In particular, the wrong action in the root state (action L in state 0; Fig. 2*F*, cyan) was preferred initially until the optimal decisions in later stages have been found. This temporal delay in computing the values of distal actions (Fig. 2*G*) is a characteristic feature of our network that we will exploit when comparing against neural and behavioral data in the following. Although neural networks implementing planning-as-inference can also result in such delayed reversals in some cases (Solway and Botvinick, 2012), they fail to compute the correct values for closed-loop control in our example task and also cannot predict reversal in this case (data not shown).

### Neural dynamics in two-alternative forced choice tasks

Having introduced the model and verified its ability to solve challenging benchmark tasks (Figs. 1–2), we considered tasks used in decision-making experiments to test our model against empirical data. We began with the simple case of two-alternative forced choice with deterministic outcomes. Padoa-Schioppa (2013) performed an experiment in which monkeys chose between two juice offers by making a saccade to one of two locations. On each trial, two types of juice (A and B, where A is preferred) were offered in different amounts. We formalized this task as consisting of a single state and two actions corresponding to the two kinds of juice being offered on a trial (Fig. 3*A*, top). Thus, our model network only consisted of two populations of neurons, each corresponding to one of the two actions, i.e., juices (Fig. 3*A*, bottom). Although the architecture of the network in this case was extremely simple, this matched the simplicity of the task it needed to solve in this special case. In general, as we show later, our model is more powerful and extends to multistep sequential tasks with potentially stochastic rewards at several steps, which imply more complex network architectures.

Despite the apparent simplicity of the task, our simulations revealed complex time courses in the activities of the model neurons that changed systematically as we varied the value of the juice encoded by a neuron (Fig. 3*B*, solid lines). First, the overall amplitude of the activity scaled with the value, which was simply attributable to the fact that the efficacies of the synapses connecting the reward input to the two units also scaled with these values. Second, and more importantly, the peak time also depended on reward magnitude, such that both medium- and high-value trials resulted in later peak times than low-value trials, despite the fact that the activity of the reward input itself followed the same time course during all trial types (Fig. 3*B*, black line). This was because for low values, the network often discovered that the encoded juice was not the best option offered, and so after a short initial increase, the activity of the neuron was actively suppressed by network interactions (compare Fig. 2*F*, dotted lines). In contrast, during high-value trials, the juice was often the best option, in which case its activity was not suppressed by the internal dynamics of the network, and primarily followed the time course of the feedforward reward input, which still continued to rise after the activity of the other neuron had been suppressed. For medium-value trials, both options had similar values, and in this case, it was the internal dynamics of the network, the mutual inhibition-driven winner-takes-all process, that took longer, resulting in a delayed peak time. These value-dependent time courses showed a close similarity to the activity of so-called “offer value” cells that Padoa-Schioppa (2013) recorded in orbitofrontal cortex (OFC; Fig. 3*B*, dotted lines).

Neural activities similar to those found in OFC have also been described in the frontal eye fields, but with the difference that rather than decaying back to baseline, neural activities persisted at an elevated baseline level for several seconds in the trial until a saccade was executed (Roesch and Olson, 2003; Fig. 3*C*), consistent with a role in reward-based decision making (Curtis and Lee, 2010). In these experiments, one of the options (B) was always unrewarded, whereas the other (A) was always rewarded, with one of two magnitudes, as indicated by a cue on each trial. We simulated this experimental paradigm in the same network using a higher baseline firing rate and constant activity for the reward input plus an initial Gaussian “bump” (Fig. 3*C*, black line). (The additional bump might be merely epiphenomenal, attributable to a transient at the presentation of a cue, or it could have a functional role by facilitating the accuracy of decision making with increased firing rates, which become unnecessary once the values are established, at which point lower activity levels may be more beneficial for metabolic reasons.) The overall time courses of neurons were similar to those found in OFC, with one key difference: neurons coding for the unchosen option B did not show an initial increase in activity (Fig. 3*C*, purple lines). The model correctly captured these results and accounted for the immediate decrease, without a transient increase, in the activity of the B population. This was attributable to the fact that the corresponding value was zero, implying that the input to the B population never increased beyond baseline, which in turn meant that lateral inhibition from the A population dominated from the very beginning of the trial. Furthermore, the model also reproduced higher persistent activity for the larger reward in the A population (Fig. 3*C*, thick vs thin blue lines) but not in the B population (Fig. 3*C*, thick vs thin purple lines). This was a fundamental property of our network, as the activities of neurons representing a suboptimal action in it are always suppressed to baseline asymptotically, even if the input, and hence the activity of neurons representing the optimal action, is persistent.

We also examined how the magnitude of activities in our network varied as the values associated with the two offers were varied systematically (Fig. 4). We found that the firing rate of a neuron showed a nonlinear, monotonically increasing dependence on the value of the juice it encoded, such that it was zero as long as this value was inferior to that of the other option, and approximately scaled with the value when it represented the better option (Fig. 4*A*, blue). We also found that the firing rate was primarily independent of the alternative offer in the latter case (data not shown). These patterns were again qualitatively similar to the behavior of offer value cells in the OFC (Padoa-Schioppa and Assad, 2006; Fig. 4*A*, green). Although the neurons in our network computed the value of a particular action (i.e., choosing a juice), theories of decision making often also require computing the value of a state, which is the value of the best action that can be taken in a situation (Sutton and Barto, 1998). Based on our network, this could be computed as the sum of the activities of all neurons corresponding to a state (i.e., implying a simple feedforward architecture from our network to such state value-representing neurons), which thus showed a nonmonotonic dependence on offer value, such that activity increased with either of the two offers becoming increasingly valuable (Fig. 4*B*, blue). This behavior was observed in another population of OFC cells, called “chosen value” cells (Padoa-Schioppa and Assad, 2006; Fig. 4*B*, green), which have indeed been suggested to receive feedforward input from offer value cells (Padoa-Schioppa, 2013).

### Behavioral dynamics in two-alternative forced choice tasks

Behavioral measures in decision tasks have often been demonstrated to offer useful constraints on the underlying neural mechanisms (Smith and Ratcliff, 2004). Thus, we compared psychometric and chronometric curves predicted by our model with those reported in binary choice experiments. In our model, following previous work (Zhang and Bogacz, 2010), a decision was made when the difference between the cumulative spike counts of the two populations reached a predetermined threshold (Fig. 5*C*). Given the population rates in our model, we set the decision threshold to reproduce average reaction times reported experimentally (Padoa-Schioppa et al., 2006) and obtained psychometric and chronometric curves without further parameter fitting (see Materials and Methods).

As we saw above, the internal dynamics of the model ensured that firing rates of the two populations directly related to the values of the two offers, at least in the steady state, and so the decisions that were based on the spike counts (effectively accumulating the rates) naturally showed a preference for the choice with the higher value. Despite decisions depending deterministically on the spike counts in the model, this preference was not deterministic because of the stochastic spiking of the neurons, the finite size of the network, and the finite reaction times enforced by the fixed decision threshold. Thus, the model naturally reproduced the standard sigmoidal psychometric curves found experimentally (Padoa-Schioppa et al., 2006; Fig. 5*A*,*B*). Both simulated and experimentally measured psychometric curves had an indifference point where the values of the two offers were equal, by definition. However, more importantly, the slopes of the psychometric curves were also well matched by our simulations without specific parameter tuning.

Reaction time data provided a particularly useful basis for comparing decision dynamics in the model to experimental data. As we described above (Fig. 3*B*), easier decisions led to the losing population being suppressed sooner, and so the spike count difference between the winning and losing populations also reached the decision threshold sooner (Fig. 5*C*). Specifically, decision times increased with the value ratio (defined as the ratio of the smaller to the larger value and thus ranging from zero to one; Fig. 5*D*, right, colored symbols), such that pooling across trials with diverse value ratios resulted in a near-lognormal distribution of decision times (Fig. 5*D*, right, gray histogram), as often found in binary choice experiments (Luce, 1986). This meant that the transformation ensuring that normalized reaction times were distributed as a standard normal was very close to logarithmic (Fig. 5*D*, left). In turn, on average, normalized reaction times were a simple linear function of the value ratio (slope = 1.63 ± 0.15, *R*^2^ = 0.958), in agreement with experimental results (slope = 1.75 ± 0.15, *R*^2^ = 0.951; Padoa-Schioppa et al., 2006; Fig. 5*E*). The residuals of this linear regression were also similarly distributed in the model and in the experimental data (Fig. 5*E*, inset). These results were again obtained without specifically fitting model parameters to these data. Furthermore, they could not be explained solely by the different inputs that the two populations received in our model. This could be seen in a simple feedforward variant of our network lacking lateral inhibition that could actively suppress the responses of neurons representing the less valuable option. (Note that this variant still implied lateral inhibition between the notional units accumulating the spike counts of neurons in our network because the final decision depended on the difference between these cumulative spike counts.) Such a feedforward network produced overlong reaction times for difficult choices, i.e., value ratios near 1, resulting in a significantly higher slope of the normalized reaction time-value ratio regression (2.22 ± 0.13). Thus, the reaction times we obtained were diagnostic of the specific recurrent interactions in our network.

### Sequential tasks

We next turned to tasks involving multiple steps as a more general test bed of our theory. In one such sequential task, monkeys were trained to produce a series of hand movements instructed by visual stimuli (Sohn and Lee, 2007). The sequence and its length, which were manipulated across trials, were determined by the first stimulus, based on which the whole sequence could thus be planned in advance. At each step of a trial, the current position and the next visual target were displayed. Executing the correct action, the hand movement taking the cursor to the visual target, brought the animal one step closer to the reward. If an incorrect action was performed, the trial was aborted without reward, and the same sequence of targets was presented again in the next trial (Fig. 6*A*, inset). (Although the presentation of the next target in each step provided some means to solve the task in a piecemeal fashion by individual one-step decisions, the neural responses and reaction times indicated that the monkeys were planning ahead, using a sequential decision-making strategy.) During the execution of the task, the activity of neurons was recorded in the pre-SMA. Pre-SMA is a frontal area strongly connected to PFC but not to primary motor cortex and involved in planning of internally generated action sequences (Nachev et al., 2008). In particular, Sohn and Lee (2007) found neurons that besides encoding kinematic parameters of the current movement also modulated their activity according to the NRMs in a trial (Fig. 6*A*).

We simulated the sequential task such that states corresponded to steps in a trial, and we report here the activity of neurons representing the optimal action in one of the states while varying the NRMs, i.e., the number of steps (movements) needed from this state to reach the reward, across trials (Fig. 6*B*). Although the activity profile of the reward input remained the same across steps (Fig. 6*B*, black line), the activities of neurons representing the correct current action showed systematic modulation by NRMs in close correspondence with the experimental data (Fig. 6*B*, colored lines). In particular, activities became larger and peaked earlier as the reward became more proximal (decreasing NRMs). Whereas the overall shape of activities was, to a large extent, inherited from our particular choice for the activity profile of the reward input, and NRM-dependent initial firing rates, these modulations of the amplitude and delay were fundamental predictions of our model. The amplitude was modulated as a result of the discounting of reward, and the response peak was delayed because activity had to spread from the reward input via neurons coding for the remaining movements to the neuron coding for the current action. These modulations matched experimental data remarkably well (compare Fig. 6*A*). Moreover, using the same mapping from neural activities to behavioral output in the model as in the simple one-step binary decision tasks described above (with a temporally discounted decision threshold; see Materials and Methods), the model also reproduced the approximately linear scaling of reaction times with NRMs (Fig. 6*D*).

The NRM-dependent modulation of delays was a hallmark of the particular dynamics our network used to compute the correct action. Specifically, the alternative proposal that the cortex implements planning as inference (Solway and Botvinick, 2012; see also Materials and Methods) did not produce such delay modulations, only modulations of the amplitude (Fig. 6*E*). Even these amplitude modulations for planning-as-inference were unlike those found experimentally as, in contrast to experimental data, the activity profiles for zero and one NRM almost completely overlapped.

Another difference between planning-as-inference and our network, which remains to be tested, was the sensitivity to the number of available actions in the current state. Our network produced the same firing rate time courses regardless of the number of actions out of which it had to choose the correct one (Fig. 6*C*). This was because incorrect actions did not yield reward, and so the corresponding neurons in our model were always inhibited and never active. That is, increasing the number of available actions with negligible value in our model amounted to adding neurons that were virtually inactive and hence had no way to influence network dynamics. (However, for the same reason, additional actions with non-negligible values did influence neural dynamics and increased behavioral reaction times in our model; data not shown.) In contrast, planning-as-inference was inherently more sensitive to this manipulation, showing strong amplitude modulations by the number of available actions such that more actions led to smaller responses (Fig. 6*F*).

Our theory also generalizes for tasks with many rewards at various time points rather than just a single reward solely at the end of a trial as in the experiments described above. To make predictions for the more general case, we simulated a modified version of the task of Figure 6 by adding an intermediate reward and by setting up subsequent wrong actions to lead to the state immediately thereafter (Fig. 7*A*). This latter change of the transition structure of the task was introduced to discourage multiple collections of the intermediate reward by simply looping between the beginning of the sequence and the immediate reward without ever progressing to the second half of the sequence. We verified that this change alone, without including the intermediate reward, did not change the predictions of Figure 6 (data not shown). Thus, any effects reported below in Figure 7 were primarily attributable to the inclusion of the intermediate reward.

In contrast to the single-reward case (Fig. 6*B*), the simulations with intermediate reward showed a nonmonotonic relationship between the peak amplitude of firing rate curves and the NRMs (Fig. 7*B*). According to our theory, neural activity encoded value (of a particular action) and not the NRMs. Although the task in Figure 6 was inappropriate for distinguishing between these two alternatives, a task such as the one proposed here enables a stronger test of our theory. Note that although the structure of transitions between states 7 and 4 matched that between states 3 and 0, the corresponding neural activities predicted by the model did not overlap, showing substantial differences in both peak rates and times (Fig. 7*B*). In particular, the activity profiles for earlier states were always higher than in the respective late states (e.g., NRM 4 vs 0 or 5 vs 1). This asymmetry was attributable to the contribution of the final reward to the values of the earlier states (albeit in a temporally discounted form), whereas the intermediate reward could not possibly contribute to the values of the later states. As reaction times in our model were directly linked to the magnitudes of neural activities (see above), we similarly predict a nonmonotonic relationship between NRM and reaction times for such multireward tasks (Fig. 7*C*).

### Multigoal environments and devaluation

Finally, we turn to a sequential task that involves choosing between multiple rewards (Fig. 8*A*). This task has been introduced by Niv et al. (2006) to study shifts in motivational state and, in particular, when the value of one choice (cheese in Fig. 8*A*, top left) depends on the current motivational state of the animal such that devaluation decreases it from a high baseline to a lower level. This task corresponds to a simple decision tree with three states (Fig. 8*A*, colored numbers), in each of which two alternative actions are available: turning left (L) or right (R). Figure 8*B* shows the evolution of neural activities in the corresponding neural network with six (groups of) neurons before devaluation. Note that, as before (Fig. 2), the activity of the neuron encoding the wrong action in the root state (state 0, action R, dark blue) is initially higher than the activity of the neuron encoding the correct action in the same state (L, light blue), and it takes time for the network to reverse this and thus arrive at representing the correct course of action. Figure 8*C* shows that the optimal sequence (L–L) is selected most of the time, and Figure 8*D* reveals again an increase in reaction times with NRMs (state 0 vs 1 and 2; compare Fig. 6*D*), as well as with the value ratio of available actions (state 1 vs 2; compare Fig. 5*E*).

The effect of devaluation can be captured in our model by simply reducing the reward value associated with the formerly preferred cheese from *r* = 4 to 2. Note that this change directly affects only the feedforward reward input into the neuron coding for action L in state 1. Nevertheless, during planning, the impact of this local change propagates to the neurons coding for the distal root state (Fig. 8*F*), yielding a reversal in choice (Fig. 8*G*) immediately after devaluation (i.e., without having experienced traversing the maze in this devalued state), which is a hallmark of goal-directed decision making. In addition, the reaction time in state 1 for the now less appealing cheese increases (Fig. 8*H*).

This task also offers a further opportunity to distinguish our model from other potential proposals for the neural network dynamics underlying goal-directed decision making. In particular, a broad class of models uses spreading activation back from goal(s) to the current state, not unlike the spreading of value signals in our network (Schmajuk and Thieme, 1992; Hasselmo, 2005; Koene and Hasselmo, 2005; Martinet et al., 2011). In these models, backward spreading of activity establishes a pattern of population activities, such that the steady-state firing rate of each cell in the network depends inversely on the distance between the state it represents and the goal state (Fig. 8*E*, gray scale), and so the animal simply needs to follow the gradient of this steady-state activity map to reach the desired goal. However, when multiple potential goals exist, each offering different rewards as in our case, this simplistic mechanism can easily fail to find the best sequence of actions (Fig. 8*E*, green trajectory). This is because spreading activation, as a fundamentally diffusive process, combines rewards in different states linearly, whereas the computation of optimal values requires nonlinear operations (Eq. 2). In contrast, in our network, lateral inhibition between neurons encoding different actions in the same state combined with the nonlinear activation function of individual neurons provides just the right nonlinearities for computing optimal values. More specifically, in our example task, simple spreading activation results in the activities of the two smaller rewards reachable from state 2 adding up such that the gradient at the root state points to the right instead of the left, despite the fact that only one of the two smaller reward items can be consumed there. In contrast, in our network, as the decision between alternative actions is being made in each state, activity caused by suboptimal targets is suppressed, and only the activity of the best (reachable) goal spreads further to downstream neurons (eventually implementing the highly nonlinear max operation).

## Discussion

Optimal decision making in a complex world is a challenging computational task in itself (Sutton and Barto, 1998), without constraining computations to be performed in a biologically plausible manner, which was the problem we addressed here. Neural network instantiations had been suggested for model-free reinforcement learning using gradient-based methods (Frémaux et al., 2010; Legenstein et al., 2010; Friedrich et al., 2011, 2014; Friedrich and Senn, 2012), as well as temporal-difference methods (Potjans et al., 2011; Frémaux et al., 2013). However, here we suggested, to our knowledge, the first biologically realistic implementation for complex model-based decisions. For biological plausibility, we used a canonical, phenomenological single-neuron model, the spike–response model (Gerstner et al., 2014), or, equivalently, the generalized linear model (Pillow et al., 2008; which has been demonstrated to be an accurate predictor of neural responses in a wide variety of brain areas), and a synaptic plasticity rule that was local and Hebbian in nature and was based on prediction errors (Gläscher et al., 2010) to acquire an appropriate internal model of the environment. We showed that model circuits constructed from such elements achieved competent performance in model-based sequential decision making and that the neural dynamics predicted by our model were consistent with a broad range of experimental data.

We suggest that our network resides in PFC. More specifically, we hypothesize that the state–action neurons, of which the dynamics our network explicitly modeled, correspond to so-called offer value cells found in OFC (Padoa-Schioppa and Assad, 2006). Whether the NRM-modulated neurons of pre-SMA receive inputs from these neurons or implement a parallel network is unclear, but our model suggests that they should be similarly modulated by values as OFC neurons are and, conversely, OFC neurons should show similar activation patterns as pre-SMA neurons in sequential decision-making tasks. We further hypothesize that the accumulation of spike count differences underlying the final decision takes place in ventromedial prefrontal cortex, which receives input from OFC (Rushworth et al., 2012) and has been implicated in the comparison of options (Wunderlich et al., 2012; De Martino et al., 2013).

Our network was constructed such that neurons encoded (or preferred) specific state–action pairs. However, empirical data suggest that actions may be represented in an ordinal fashion, such that even the same action in the same state may be represented by different neurons depending on how distal (i.e., how many time steps away) it is to the current state of the animal (Mushiake et al., 2006). It is straightforward to extend our model to represent state–action–time step tuples by replicating the state–action neurons for each time step in our current network and having excitatory synapses connect to neurons encoding the previous time step. Whereas such an implementation allows for time-dependent policies, it deals only with the case of finite horizon and comes at the expense of growing the size of the network with time horizon. Nevertheless, based on such a representation, action–time step neurons can be obtained by projecting down into a downstream area, akin to obtaining chosen value cells from offer value cells in OFC, and thus account for ordinal action representations. To make our model metabolically more efficient, and more comparable in that regard to earlier models (Solway and Botvinick, 2012), it could further be extended such that a separate network computing the reachability of future states from the current one (e.g., by forward spread of activation) provides an extra input to our network so that only neurons representing reachable states (and actions) in it are close to the threshold.

To simplify exposition, we presented our network as a minimal neural circuit allowing neurons to have both excitatory and inhibitory synapses. The violation of Dale's principle could be avoided by explicitly considering the interneurons that mediate lateral inhibition between all excitatory cells coding for the same state (but potentially different actions). Moreover, bisynaptic inhibition may also account for offer value cells showing negative modulation by value (Padoa-Schioppa and Assad, 2006) and pre-SMA cells negatively modulated by NRM (Sohn and Lee, 2007), which our simplified network could not capture.

Lateral inhibition was a key element of the dynamics of our network. While many previous models used lateral inhibition to implement a winner-take-all mechanism between different choices, there are important differences between those models and ours. This is because the precise ways in which lateral inhibition acts in a model, and in particular whether it acts between units having neural-like or longer time constants, can have profound consequences for its dynamics (Teodorescu and Usher, 2013). For example, models of nonsequential perceptual decision making typically use long (∼100 ms) time constants that match the time scale of individual trials to achieve reliable accumulation of evidence, whereas in our network we used shorter, more realistic membrane time constants (∼20 ms) and thus relied on accumulation happening as a “postprocessing step.” This difference has important implications for the interplay between accumulation and lateral inhibition. In earlier models, lateral inhibition occurred at the level of the accumulated decision variables (Bogacz et al., 2006; Wong and Wang, 2006). In contrast, in our model, which might otherwise appear a close (spiking) analog of these earlier models in the case of two-alternative forced choices, it affected already the nonaccumulated decision variables represented by the neurons of our network. Inhibition between accumulated variables tends to lead to unrealistically skewed reaction time distributions (Smith and Ratcliff, 2004), which our model successfully avoided (Fig. 5) but which we also found in simulations of an alternative variant of our network lacking inhibition between fast time scale neurons (data not shown). Moreover, the specific form of lateral inhibition used in our network was also crucial for allowing us to generalize it to solving the ecologically more relevant and computationally more challenging task of multistep sequential decision making (in contrast to simple spreading activation models that do not use lateral inhibition; Fig. 8*E*) and also predicted neural and behavioral data in such richer tasks with high accuracy.

As a consequence of the specific role that lateral inhibition plays in its dynamics, our model also provides an alternative account of the stochasticity of decisions and the distribution of decision times in simple perceptual decision-making tasks in which the stimulus is not explicitly stochastic. Previous models relied on stochasticity in the inputs to the network, even for nonstochastic stimuli, and the fact that this stochasticity needs to be integrated out over time (Usher and McClelland, 2001; Smith and Ratcliff, 2004). Thus, both psychometric and chronometric curves primarily depended on this external noise. In contrast, our network receives deterministic input, and so psychometric curves are a result of the spiking “noise” within the network itself, whereas chronometric curves are a consequence of the specific form of lateral inhibition used in it.

Our approach to perform sequential decision making was based on the principle of dynamic programming (Bellman, 1957): instead of performing a sequential tree search, planning in our model occurred in a near-parallel fashion, as suggested by near-parallel neural activations observed in PFC (Averbeck et al., 2002; Mushiake et al., 2006). Importantly, dynamic programming-based, goal-directed decision-making algorithms, such as that implemented by our network, require an internal model of task contingencies that needs to be acquired through interactions and experience with the environment. For this, our network required that the same neurons that encode states and actions during planning of an action sequence become activated later while that sequence is being performed, so that the synaptic weights between neurons faithfully reflect the transition and reward probabilities implied by the task. Such reactivation of neurons taking part in planning and execution has also been observed in the PFC (Mushiake et al., 2006).

Off-line replay of experience, during periods of rest or sleep, as observed throughout the neocortex (Hoffman and McNaughton, 2002) and, in particular, in multiple brain areas implicated in goal-directed decision making, such as the ventral striatum (Pennartz et al., 2004; Lansink et al., 2008), the PFC (Euston et al., 2007; Peyrache et al., 2009), and the hippocampus (Foster and Wilson, 2006; Gupta et al., 2010), may also serve to reinforce and consolidate internal models of the environment (Lin, 1992; Mnih et al., 2015). Although several other models have been proposed in which the replay of experience underlies model learning (Redish and Touretzky, 1998), our model differs from these in a crucial aspect. Although those models used Hebbian plasticity to store information about the experienced sequences, our model requires either the replay of sequences in reverse temporal order or forward replay to be coupled with anti-Hebbian forms of plasticity (such that post-before-presynaptic activation is needed for potentiation). This is because in our model, an excitatory synaptic weight connecting a presynaptic neuron *i* to postsynaptic neuron *j* represents the probability of reaching the state represented by neuron *i* from that represented by neuron *j*. Reverse replay has only been observed in the hippocampus (Foster and Wilson, 2006; Diba and Buzsáki, 2007), and it remains to be tested whether, for example, it also exists in PFC or whether there are anti-Hebbian forms of plasticity operating there.

On-line reverse hippocampal replay in the form of spreading activation has also been suggested to provide the neural substrate of spatial navigation (Hasselmo, 2005; Martinet et al., 2011). Considering navigation a special case of sequential decision making allows a direct comparison between these models and ours. In line with previous work, our model predicts activity spreading from neurons representing the goal to neurons representing more proximal locations. However, the precise form of spreading activation in our network is different, and notably nonlinear, thus allowing the network to solve the more general problem of maximizing return in sequential decision-making tasks with multiple rewards, for which classical spreading activation models would fail to account (Fig. 8). Therefore, our model also makes the novel prediction that the replay-like phenomenon of spreading activation during planning should also generalize to distinctly nonspatial domains (albeit perhaps in other cortical areas).

Previous proposals for how cortical circuits may solve sequential decision tasks were based on the powerful idea of using probabilistic inference algorithms for planning (Attias, 2003; Toussaint and Storkey, 2006). Although this idea is conceptually and algorithmically attractive, especially in light of the converging evidence that cortical circuits may naturally perform probabilistic inference (Fiser et al., 2010), the neural instantiations suggested so far relied on two particularly speculative assumptions (Solway and Botvinick, 2012): they required multiplicative interactions between presynaptic neurons and assumed that dendrites approximately perform a logarithmic transformation on their inputs. Furthermore, state and action neurons needed to be replicated for each time step of a sequential task; hence, the size of the network grew with the time horizon, which had to be finite. Moreover, as our illustrative example task revealed (Fig. 2), the particular form of probabilistic inference afforded in these networks (belief propagation) leads to severely suboptimal behavior in even simple test cases, which our network successfully solved. Whereas belief propagation has been often evoked as the algorithmic basis of how the cortex performs probabilistic inference (Lochmann and Deneve, 2011), recent results suggest that the cortex may, in fact, implement other kinds of inference algorithms (Fiser et al., 2010; Berkes et al., 2011; Buesing et al., 2011; Hennequin et al., 2014) that are rich enough to capture the structure of any decision-making task. Thus, even simple tasks such as those presented here could be used to discriminate competing proposals for the type of algorithm that the cortex implements for model-based decision making.

## Notes

Supplemental material for this article, which consists of python scripts used for the simulations, the proof that the dynamics of our network converge to a fixed point where neural activities come to represent the optimal values that satisfy the Bellman optimality equation, as well as additional information, is available at https://github.com/j-friedrich/Goal-directed_decision_making_with_spiking_neurons. This material has not been peer reviewed.

## References

[B1] Attias H (2003). Planning by probabilistic inference. http://research.microsoft.com/en-us/um/cambridge/events/aistats2003/proceedings/papers.htm.

[B2] Averbeck BB, Chafee MV, Crowe DA, Georgopoulos AP (2002). Parallel processing of serial movements in prefrontal cortex. Proc Natl Acad Sci U S A.

[B3] Bellman R (1957). Dynamic programming.

[B4] Berkes P, Orbán G, Lengyel M, Fiser J (2011). Spontaneous cortical activity reveals hallmarks of an optimal internal model of the environment. Science.

[B5] Bogacz R, Brown E, Moehlis J, Holmes P, Cohen JD (2006). The physics of optimal decision making: a formal analysis of models of performance in two-alternative forced choice tasks. Psychol Rev.

[B6] Botvinick M, An J (2009). Goal-directed decision making in prefrontal cortex: a computational framework. Adv Neural Inf Process Syst.

[B7] Botvinick M, Toussaint M (2012). Planning as inference. Trends Cogn Sci.

[B8] Buesing L, Bill J, Nessler B, Maass W (2011). Neural dynamics as sampling: a model for stochastic computation in recurrent networks of spiking neurons. PLoS Comput Biol.

[B9] Curtis CE, Lee D (2010). Beyond working memory: the role of persistent activity in decision making. Trends Cogn Sci.

[B10] Daw ND, Niv Y, Dayan P (2005). Uncertainty-based competition between prefrontal and dorsolateral striatal systems for behavioral control. Nat Neurosci.

[B11] Daw ND, Gershman SJ, Seymour B, Dayan P, Dolan RJ (2011). Model-based influences on humans' choices and striatal prediction errors. Neuron.

[B12] Dearden R, Friedman N, Russell S (1998). Bayesian Q-learning.

[B13] Deisenroth M, Rasmussen CE, Peters J (2009). Gaussian process dynamic programming. Neurocomputing.

[B14] De Martino B, Fleming SM, Garrett N, Dolan RJ (2013). Confidence in value-based choice. Nat Neurosci.

[B15] Diba K, Buzsáki G (2007). Forward and reverse hippocampal place-cell sequences during ripples. Nat Neurosci.

[B16] Euston DR, Tatsuno M, McNaughton BL (2007). Fast-forward playback of recent memory sequences in prefrontal cortex during sleep. Science.

[B17] Fiser J, Berkes P, Orbán G, Lengyel M (2010). Statistically optimal perception and learning: from behavior to neural representations. Trends Cogn Sci.

[B18] Foster DJ, Wilson MA (2006). Reverse replay of behavioural sequences in hippocampal place cells during the awake state. Nature.

[B19] Frémaux N, Sprekeler H, Gerstner W (2010). Functional requirements for reward-modulated spike-timing-dependent plasticity. J Neurosci.

[B20] Frémaux N, Sprekeler H, Gerstner W (2013). Reinforcement learning using a continuous time actor-critic framework with spiking neurons. PLoS Comput Biol.

[B21] Friedrich J, Senn W (2012). Spike-based decision learning of Nash equilibria in two-player games. PLoS Comput Biol.

[B22] Friedrich J, Urbanczik R, Senn W (2010). Learning spike-based population codes by reward and population feedback. Neural Comput.

[B23] Friedrich J, Urbanczik R, Senn W (2011). Spatio-temporal credit assignment in neuronal population learning. PLoS Comput Biol.

[B24] Friedrich J, Urbanczik R, Senn W (2014). Code-specific learning rules improve action selection by populations of spiking neurons. Int J Neural Syst.

[B25] Gerstner W, Kistler W, Naud R, Paninski L (2014). Neuronal dynamics: from single neurons to networks and models of cognition.

[B26] Gläscher J, Daw N, Dayan P, O'Doherty JP (2010). States versus rewards: dissociable neural prediction error signals underlying model-based and model-free reinforcement learning. Neuron.

[B27] Gupta AS, van der Meer MA, Touretzky DS, Redish AD (2010). Hippocampal replay is not a simple function of experience. Neuron.

[B28] Hasselmo ME (2005). A model of prefrontal cortical mechanisms for goal-directed behavior. J Cogn Neurosci.

[B29] Hennequin G, Aitchison L, Lengyel M (2014). Fast sampling-based inference in balanced neuronal networks. Adv Neural Inf Process Syst.

[B30] Hoffman KL, McNaughton BL (2002). Coordinated reactivation of distributed memory traces in primate neocortex. Science.

[B31] Jolivet R, Rauch A, Lüscher HR, Gerstner W (2006). Predicting spike timing of neocortical pyramidal neurons by simple threshold models. J Comput Neurosci.

[B32] Kearns M, Singh S (1999). Finite-sample convergence rates for Q-learning and indirect algorithms. Adv Neural Inf Process Syst.

[B33] Keramati M, Dezfouli A, Piray P (2011). Speed/accuracy trade-off between the habitual and the goal-directed processes. PLoS Comput Biol.

[B34] Koene RA, Hasselmo ME (2005). An integrate-and-fire model of prefrontal cortex neuronal activity during performance of goal-directed decision making. Cereb Cortex.

[B35] Koller D, Friedman N (2009). Probabilistic graphical models: principles and techniques.

[B36] Lansink CS, Goltstein PM, Lankelma JV, Joosten RN, McNaughton BL, Pennartz CM (2008). Preferential reactivation of motivationally relevant information in the ventral striatum. J Neurosci.

[B37] Lauritzen SL, Spiegelhalter DJ (1988). Local computations with probabilities on graphical structures and their application to expert systems. J R Stat Soc Ser B Stat Methodol.

[B38] Legenstein R, Chase SM, Schwartz AB, Maass W (2010). A reward-modulated Hebbian learning rule can explain experimentally observed network reorganization in a brain control task. J Neurosci.

[B39] Lin LJ (1992). Self-improving reactive agents based on reinforcement learning, planning and teaching. Mach Learn.

[B40] Lochmann T, Deneve S (2011). Neural processing as causal inference. Curr Opin Neurobiol.

[B41] Luce RD (1986). Response times.

[B42] Martinet LE, Sheynikhovich D, Benchenane K, Arleo A (2011). Spatial learning and action planning in a prefrontal cortical network model. PLoS Comput Biol.

[B43] McCormick DA, Connors BW, Lighthall JW, Prince DA (1985). Comparative electrophysiology of pyramidal and sparsely spiny stellate neurons of the neocortex. J Neurophysiol.

[B44] Mnih V, Kavukcuoglu K, Silver D, Rusu AA, Veness J, Bellemare MG, Graves A, Riedmiller M, Fidjeland AK, Ostrovski G, Petersen S, Beattie C, Sadik A, Antonoglou I, King H, Kumaran D, Wierstra D, Legg S, Hassabis D (2015). Human-level control through deep reinforcement learning. Nature.

[B45] Mushiake H, Saito N, Sakamoto K, Itoyama Y, Tanji J (2006). Activity in the lateral prefrontal cortex reflects multiple steps of future events in action plans. Neuron.

[B46] Nachev P, Kennard C, Husain M (2008). Functional role of the supplementary and pre-supplementary motor areas. Nat Rev Neurosci.

[B47] Niv Y, Joel D, Dayan P (2006). A normative perspective on motivation. Trends Cogn Sci.

[B48] O'Doherty J, Dayan P, Schultz J, Deichmann R, Friston K, Dolan RJ (2004). Dissociable roles of ventral and dorsal striatum in instrumental conditioning. Science.

[B49] Padoa-Schioppa C (2013). Neuronal origins of choice variability in economic decisions. Neuron.

[B50] Padoa-Schioppa C, Assad JA (2006). Neurons in the orbitofrontal cortex encode economic value. Nature.

[B51] Padoa-Schioppa C, Jandolo L, Visalberghi E (2006). Multi-stage mental process for economic choice in capuchins. Cognition.

[B52] Papadimitriou CH, Tsitsiklis JN (1987). The complexity of Markov decision processes. Math Oper Res.

[B53] Pennartz CM, Lee E, Verheul J, Lipa P, Barnes CA, McNaughton BL (2004). The ventral striatum in off-line processing: ensemble reactivation during sleep and modulation by hippocampal ripples. J Neurosci.

[B54] Peyrache A, Khamassi M, Benchenane K, Wiener SI, Battaglia FP (2009). Replay of rule-learning related neural patterns in the prefrontal cortex during sleep. Nat Neurosci.

[B55] Pfister JP, Dayan P, Lengyel M (2010). Synapses with short-term plasticity are optimal estimators of presynaptic membrane potentials. Nat Neurosci.

[B56] Pillow JW, Paninski L, Uzzell VJ, Simoncelli EP, Chichilnisky EJ (2005). Prediction and decoding of retinal ganglion cell responses with a probabilistic spiking model. J Neurosci.

[B57] Pillow JW, Shlens J, Paninski L, Sher A, Litke AM, Chichilnisky EJ, Simoncelli EP (2008). Spatio-temporal correlations and visual signalling in a complete neuronal population. Nature.

[B58] Potjans W, Diesmann M, Morrison A (2011). An imperfect dopaminergic error signal can drive temporal-difference learning. PLoS Comput Biol.

[B59] Rao RP (2004). Hierarchical Bayesian inference in networks of spiking neurons. Adv Neural Inf Process Syst.

[B60] Redish AD, Touretzky DS (1998). The role of the hippocampus in solving the Morris water maze. Neural Comput.

[B61] Resulaj A, Kiani R, Wolpert DM, Shadlen MN (2009). Changes of mind in decision-making. Nature.

[B62] Rigotti M, Barak O, Warden MR, Wang XJ, Daw ND, Miller EK, Fusi S (2013). The importance of mixed selectivity in complex cognitive tasks. Nature.

[B63] Roesch MR, Olson CR (2003). Impact of expected reward on neuronal activity in prefrontal cortex, frontal and supplementary eye fields and premotor cortex. J Neurophysiol.

[B64] Rushworth MF, Kolling N, Sallet J, Mars RB (2012). Valuation and decision-making in frontal cortex: one or many serial or parallel systems?. Curr Opin Neurobiol.

[B65] Savin C, Dayan P, Lengyel M (2014). Optimal recall from bounded metaplastic synapses: predicting functional adaptations in hippocampal area CA3. PLoS Comput Biol.

[B66] Schmajuk NA, Thieme AD (1992). Purposive behavior and cognitive mapping: a neural network model. Biol Cybern.

[B67] Schultz W, Dayan P, Montague PR (1997). A neural substrate of prediction and reward. Science.

[B68] Seymour B, O'Doherty JP, Dayan P, Koltzenburg M, Jones AK, Dolan RJ, Friston KJ, Frackowiak RS (2004). Temporal difference models describe higher-order learning in humans. Nature.

[B69] Smith PL, Ratcliff R (2004). Psychology and neurobiology of simple decisions. Trends Neurosci.

[B70] Sohn JW, Lee D (2007). Order-dependent modulation of directional signals in the supplementary and presupplementary motor areas. J Neurosci.

[B71] Solway A, Botvinick MM (2012). Goal-directed decision making as probabilistic inference: a computational framework and potential neural correlates. Psychol Rev.

[B72] Strait CE, Blanchard TC, Hayden BY (2014). Reward value comparison via mutual inhibition in ventromedial prefrontal cortex. Neuron.

[B73] Strens M (2000). A Bayesian framework for reinforcement learning.

[B74] Sutton RS, Barto AG (1998). Reinforcement learning: an introduction.

[B75] Teodorescu AR, Usher M (2013). Disentangling decision models: from independence to competition. Psychol Rev.

[B76] Toussaint M, Storkey A (2006). Probabilistic inference for solving discrete and continuous state Markov decision processes.

[B77] Truccolo W, Eden UT, Fellows MR, Donoghue JP, Brown EN (2005). A point process framework for relating neural spiking activity to spiking history, neural ensemble, and extrinsic covariate effects. J Neurophysiol.

[B78] Usher M, McClelland JL (2001). The time course of perceptual choice: the leaky, competing accumulator model. Psychol Rev.

[B79] Wong KF, Wang XJ (2006). A recurrent network mechanism of time integration in perceptual decisions. J Neurosci.

[B80] Wunderlich K, Dayan P, Dolan R (2012). Mapping value based planning and extensively trained choice in the human brain. Nat Neurosci.

[B81] Zhang J, Bogacz R (2010). Optimal decision making on the basis of evidence represented in spike trains. Neural Comput.

